# Better Together: Ilmenite/Hematite Junctions for Photoelectrochemical Water Oxidation

**DOI:** 10.1021/acsami.0c12275

**Published:** 2020-09-28

**Authors:** Serena Berardi, Jagadesh Kopula Kesavan, Lucia Amidani, Elia Marek Meloni, Marcello Marelli, Federico Boscherini, Stefano Caramori, Luca Pasquini

**Affiliations:** † Department of Chemical and Pharmaceutical Sciences, University of Ferrara, via Luigi Borsari 46, 44121 Ferrara, Italy; ‡ Department of Physics and Astronomy, Alma Mater Studiorum−Università di Bologna, viale Carlo Berti Pichat 6/2, 40127 Bologna, Italy; § Helmholtz-Zentrum Dresden-Rossendorf, c/o European Synchrotron Radiation Facility, 71 Avenue des Martyrs, 38000 Grenoble, France; ∥ CNR-SCITEC, Istituto di Scienze e Tecnologie Chimiche “Giulio Natta”, Via Gaudenzio Fantoli 16/15, 20138 Milano, Italy

**Keywords:** photoelectrochemistry, hematite, titanium, EXAFS, electron microscopy, transient absorption spectroscopy, electrochemical impedance spectroscopy, heterointerface, oxygen evolution catalyst

## Abstract

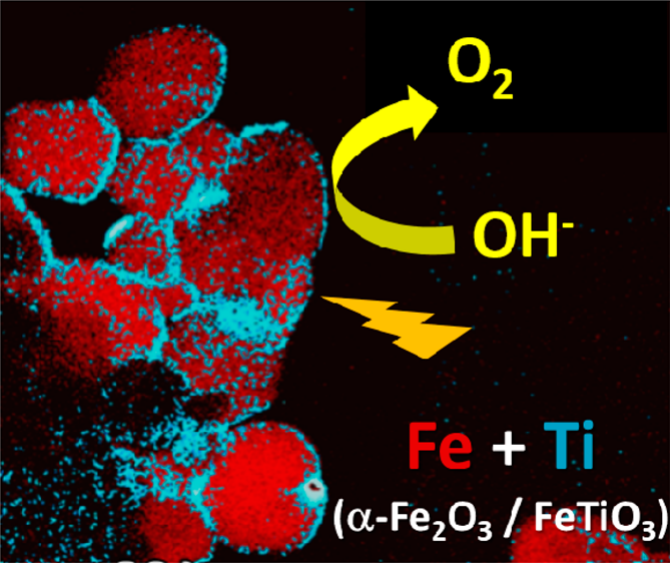

Hematite (α-Fe_2_O_3_) is an earth-abundant indirect n-type semiconductor displaying a band gap of about 2.2 eV, useful for collecting a large fraction of visible photons, with frontier energy levels suitably aligned for carrying out the photoelectrochemical water oxidation reaction under basic conditions. The modification of hematite mesoporous thin-film photoanodes with Ti(IV), as well as their functionalization with an oxygen-evolving catalyst, leads to a 6-fold increase in photocurrent density with respect to the unmodified electrode. In order to provide a detailed understanding of this behavior, we report a study of Ti-containing phases within the mesoporous film structure. Using X-ray absorption fine structure and high-resolution transmission electron microscopy coupled with electron energy loss spectroscopy, we find that Ti(IV) ions are incorporated within ilmenite (FeTiO_3_) near-surface layers, thus modifying the semiconductor–electrolyte interface. To the best of our knowledge, this is the first time that an FeTiO_3_/α-Fe_2_O_3_ composite is used in a photoelectrochemical setup for water oxidation. In fact, previous studies of Ti(IV)-modified hematite photoanodes reported the formation of pseudobrookite (Fe_2_TiO_5_) at the surface. By means of transient absorption spectroscopy, transient photocurrent experiments, and electrochemical impedance spectroscopy, we show that the formation of the Fe_2_O_3_/FeTiO_3_ interface passivates deep traps at the surface and induces a large density of donor levels, resulting in a strong depletion field that separates electron and holes, favoring hole injection in the electrolyte. Our results provide the identification of a phase coexistence with enhanced photoelectrochemical performance, allowing for the rational design of new photoanodes with improved kinetics.

## 1 Introduction

The need to find solutions to the global energy crisis has prompted the scientific community to propose various approaches aimed at the exploitation of renewable energy sources. Among them, solar energy conversion in the form of chemical energy (so-called artificial photosynthesis) can be effectively realized in photoelectrochemical cells (PECs). In such devices, the illumination of semiconductor-based materials triggers several complex charge separation processes, leading to the formation of energy-rich molecules, also known as solar fuels.^1,2^ One of the most investigated alternative fuels, hydrogen, can be obtained at the cathodic side of a PEC system for photoinduced water splitting, while at the anodic side oxygen evolves.^3,4^ In order to realize optimized composite photoelectrodes it is of paramount importance to gain a thorough understanding of the physical properties of commonly used materials as well as to investigate new ones.

In particular, in this work we use advanced spectroscopic and electrochemical techniques in order to shed light on the interfacial composition and the charge-transfer dynamics of Ti(IV)-modified hematite films with enhanced PEC performance.

The photoanodic platform of choice, i.e., hematite (α-Fe_2_O_3_), is widely used in PEC-based systems,^5,6^ thanks to (i) its intermediate band gap (ca. 2.2 eV), allowing for the absorption of visible photons up to 590 nm, (ii) the valence band maximum favorably located with respect to the potential of the H_2_O/O_2_ redox couple, (iii) the essentially null toxicity, (iv) the good chemical stability in aqueous alkaline media, and (v) its abundance, leading to low cost. However, the photoelectrochemical performance of hematite is limited by the low mobility of charge carriers and the slow hole transfer to the electrolyte.^5^ These detrimental aspects are generally addressed using strategies involving doping, nanostructuring, and the functionalization with catalytic layers, or a combination thereof.^5^

In our work, Ti(IV) was chosen as the dopant agent since it is reported to increase the electron density and the overall conductivity of the resulting material,^7^ as well as to passivate hematite surface states.^7,8^ All these aspects contribute to extend the lifetime of the charge carriers, reducing their recombination and improving the photocurrent. However, the actual origin of the Ti-induced improvement of the PEC performance remains elusive to date. We thus use X-ray absorption fine structure (XAFS) and high-resolution transmission electron microscopy (HRTEM) as powerful tools to study the local atomic and electronic structure of condensed matter. Detailed analysis of the fine structure oscillations in the extended energy range (the so-called EXAFS: extended X-ray absorption fine structure) based on the real-space multiple scattering formalism is able to provide a quantitative determination of the composition of the first few coordination shells around the excited atom, the interatomic distances, and their spread around the average value. Moreover, analysis of the line shape of the spectral region near the absorption edge (so-called XANES: X-ray absorption near-edge structure) can provide important information on the oxidation state, valence, atomic geometry, and site/symmetry selected density of states (DOS) of unoccupied electronic states. Due to its local character, XAFS is particularly useful to study disordered or defective atomic arrangements in condensed matter, including especially thin films. Also very important for the present investigation, XAFS is the premier structural tool to determine the incorporation site of dopants in condensed matter since it is possible in many cases to isolate the XAFS cross section of the excited atom from the background contribution.^9−11^ The objectives of the XAFS measurements in the present case were to assess the possible modification of the hematite matrix upon addition of Ti and to determine the incorporation sites. Concerning the second point, we sought to determine whether Ti is found in a local structure reminiscent of one of the titanium oxides, as an isolated Ti impurity substitutional to Fe, or in ilmenite, an Fe–Ti mixed oxide, or in any other form. The spatial distribution of Ti-containing phases within the mesoporous thin films was determined by TEM equipped with electron energy loss spectroscopy (EELS), which provides information complementary to XAFS together with the high spatial resolution typical of TEM.

At the same time, we employ transient absorption spectroscopy (TAS), transient photocurrent (TPC) experiments, and electrochemical impedance spectroscopy (EIS), in order to evaluate the effect of the interfacial modifications on the collection and recombination dynamics of the photogenerated charges, involving the participation of different kinds of surface states. It has recently been pointed out that this is a critical (and often overlooked) issue for the operation of hematite-based photoanodes for PEC water oxidation, which can be usefully engineered and exploited.^12^

Our work thus provides spectroscopic and photoelectrochemical insights enabling us to unravel the role of Ti(IV) and catalytic layers on the performance of composite hematite electrodes, as well as to rationally design new photoanodes with optimized kinetics.

## 2 Experimental Methods

The photoanodes were prepared by depositing mesoporous hematite thin films (300–400 nm thick, indicated as MPH) on fluorine-doped tin oxide (FTO) slides following an easy-to-scale electrophoretic method.^13,14^ When the preparation is made in the presence of a given amount of dissolved titanium (IV) butoxide (nominal Ti content of 5 and 10 wt %), Ti-modified MPH_5Ti and MPH_10Ti electrodes are obtained, respectively. Detailed information on the procedure is provided in the Supporting Information. Some of the MPH_5Ti photoanodes were further functionalized with an amorphous iron oxyhydroxide oxygen-evolving catalyst (FeOEC) deposited by successive ionic layer adsorption and reaction (SILAR), as previously reported in conjunction with different absorbing materials.^13,15−17^ These electrodes are named MPH_5Ti-FeOEC throughout the manuscript.

Photoelectrochemical measurements were carried out with a PGSTAT 30 electrochemical workstation in a three-electrode configuration, using a saturated calomel electrode (SCE) and a Pt wire as reference and counter electrodes, respectively. A LOT-Oriel solar simulator, equipped with an AM1.5G filter and set to 0.1 W/cm^2^ incident irradiance power, was used as the illumination source. *J*–*V* curves were recorded at 20 mV/s scan rate.

For the EIS measurements under illumination, the photoanodes were sampled in the selected potential range at 50 mV intervals, employing an FRA2.v10 frequency response analyzer controlled by software Nova 1.10. A 10 mV amplitude sinusoidal perturbation, the frequency of which ranged between 100 kHz and 0.1 Hz, was used. The EIS data were fitted by means of the equivalent circuit reported in Figure S13D using the ZView software, with typical relative errors lower than 15%.

Nanosecond TAS was carried out using pump pulses with 355 nm wavelength and ca. 500 μJ/cm^2^ fluence. The spectra were collected both under open-circuit potential *V*_OC_ and under anodic bias in a two-electrode configuration using a Pt wire as a counter electrode. TPC experiments were performed under the same excitation conditions, with the photoanodes placed in a three-compartment cell. TPC measurements were also conducted under a white light bias from a solar simulator set to 0.4 W/cm^2^. Further details on the experimental setup for transient measurements are provided in the Supporting Information.

All experiments were performed in a 0.1 KOH aqueous electrolyte (pH 13.3). Unless otherwise stated, the potential values are given versus the reversible hydrogen electrode (RHE), applying the following conversion:
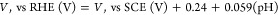
1

XAFS measurements were carried out at the Ti and Fe K edges at BM23 of the European Synchrotron Radiation Facility (ESRF), Grenoble. Measurements on Ti-modified MPH were performed in fluorescence mode using a Vortex Si drift diode detector placed in the horizontal plane at right angles to the impinging beam, at room temperature. Reference powder samples of hematite, magnetite, rutile, anatase, brookite, ilmenite, and pseudobrookite were also measured in transmission mode. The pretreatment process for all XAFS spectra was performed using the Athena program of IFEFFIT software package.^18^ The pre-edge features were fitted with Gaussian profiles using the Fityk package.^19^ The EXAFS spectra were analyzed using the Artemis code^18^ using simulated scattering paths calculated using FEFF 6.0.

TEM analyses were conducted on specimens prepared by scratching the photoanodes surface with a scalpel and collecting the powder by adherence onto a copper TEM grid coated by a holey carbon film.^20^ The employed instrument was a ZEISS LIBRA200 FE electron microscope equipped with an in-column Ω-filter analyzer. ESI (electron spectroscopy imaging) elemental maps for Ti and Fe were collected at the L_2,3_ edge (at energy loss 462 and 708 eV, respectively) by a three-windows methods. EELS spectra were collected at the Ti L_2,3_ edge in the energy loss range of 420–500 eV. Electron diffraction analysis were performed by CrysTBox software.^21^

## 3 Results and Discussion

The photoelectrochemical performances of the photoanodes, collected under 1 sun illumination (0.1 W/cm^2^, AM1.5G) in 0.1 M KOH, are reported in Figure S1A. The results confirm a 4-fold improvement of the maximum photocurrent density for the MPH_5Ti electrodes (up to 1 mA/cm^2^ at 1.67 V vs RHE) with the respect to the bare MPH. A decrease in the photoanodic performance was instead observed for a higher (or lower) Ti(IV) amount.^13^ The functionalization of the MPH_5Ti electrodes with FeOEC resulted, as expected, in a further enhancement of the PEC outcomes both in terms of onset (ca. 200 mV shift, Figure S1A) and of net photocurrent (up to 1.45 mA/cm^2^ at 1.67 V), thanks to improved photohole trapping in the catalyst’s reactive sites, as previously demonstrated by EIS measurements.^13,16,17^ Furthermore, by means of Mott–Schottky analysis, we have detected a 3-fold increase in the donor density for MPH_5Ti (and MPH_5Ti-FeOEC) versus the bare MPH, likely due to the formation of a junction between hematite and a Ti-containing phase.^13^ However, the exact identification of the local structure of this phase could not be determined either by X-ray photoemission spectroscopy (XPS) or by X-ray diffraction (XRD).^13^ XPS shows that the oxidation states of Fe and Ti are 3+ and 4+, respectively. However, this does not allow us to distinguish between different Ti-containing phases like TiO_2_ (rutile, anatase, or brookite), FeTiO_3_, and Fe_2_TiO_5_. In order to provide an in-depth understanding of the origin of the enhanced PEC performance of these modified photoanodes, the local structure and oxidation state of Fe and Ti have been studied by XAFS and TEM–EELS.

### 3.1 XAFS and TEM Measurements on the Photoanodes

**Figure 1:**
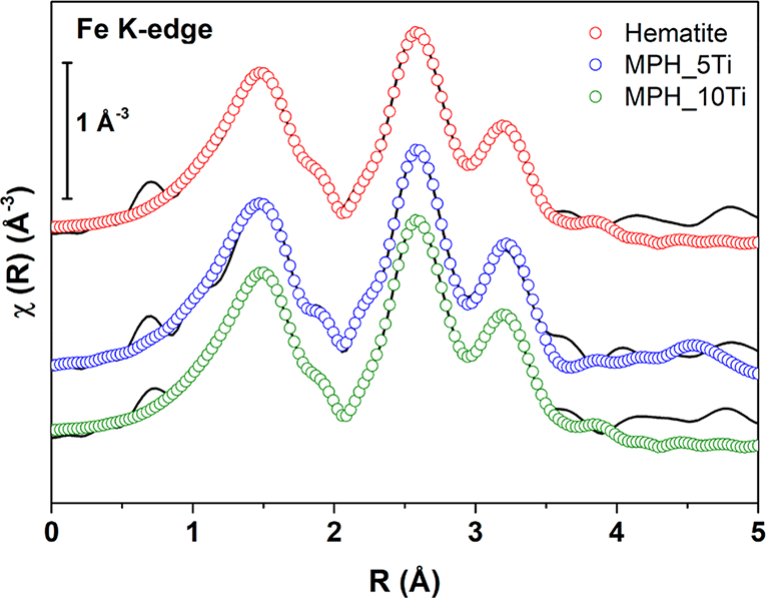
Fourier-transformed Fe K edge EXAFS (*k*^2^-weighted) spectra of Ti-modified MPH and reference hematite with their best fits (data reported as continuous line and fits as empty circles). Spectra have been vertically offset for clarity.

The background-subtracted Fe K edge EXAFS spectra of MPH_5Ti and MPH_10Ti and reference hematite are reported in Figure S2. It is clear that the spectra are all very similar to each other. Hematite has trigonal crystal structure with space group 167 (i.e., *R*3̅*c*:*H*) in which each Fe atom is found in a distorted octahedron formed by six O ones (three atoms at 1.93 Å and three at 2.08 Å). Figure 1 shows as continuous lines the Fourier transforms of the EXAFS spectra performed in the range of 3–13.9 Å^–1^. These spectra were fitted in *R*-space in the range of 1–4.8 Å using the known structure of hematite to calculate scattering paths.

The many-body amplitude reduction factor was fixed to the value obtained from a fit of a hematite reference spectrum (*S*_0_^2^ = 0.90); the fitting parameters were a common value of the energy origin shift (Δ*E*_0_) and, for each contribution listed below, the interatomic distances (*R*) and Debye–Waller factors (σ^2^). *R* goodness-of-fit factors were ∼0.01 for all samples, and the fits are reported as empty circles in Figure 1.

We found that the first peak at ∼1.5 Å and a small shoulder at ∼1.90 Å are due to the contribution from single scattering of the six first-shell O atoms, the second peak is due to one Fe atom at 2.86 Å and three Fe atoms at 2.97 Å, and the third peak is due to three Fe atoms at 3.38 Å and six Fe atoms at 3.68 Å. No multiple scattering contributions were included because of their very low amplitude. The quantitative results of the fit are listed in Table S1. The fitting parameters of Ti-modified MPH and those of reference hematite exhibit negligible differences, being both compatible with literature data for hematite;^22,23^ this indicates that the presence of Ti(IV) does not alter significantly the local structure of Fe, which remains very similar to that of hematite.

**Figure 2:**
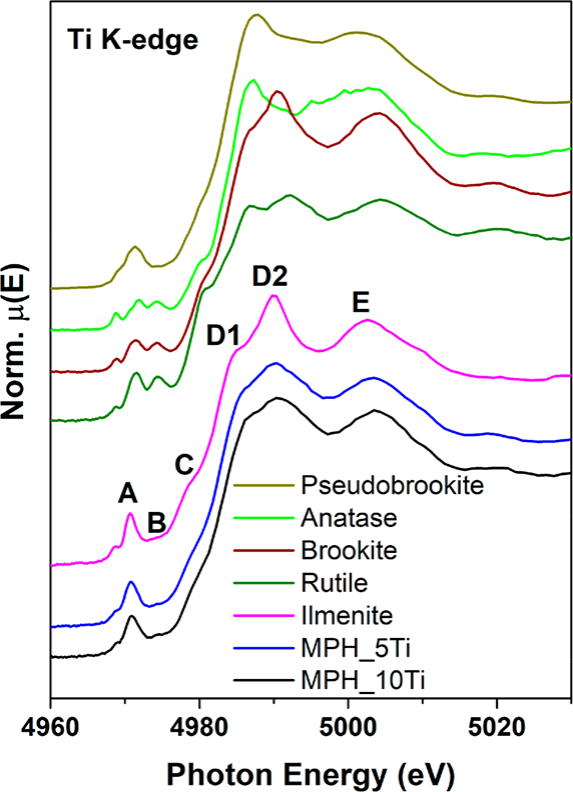
Ti K edge normalized XANES spectra of Ti-modified MPH compared to pseudobrookite, anatase, brookite, rutile, and ilmenite reference spectra.

**Figure 3:**
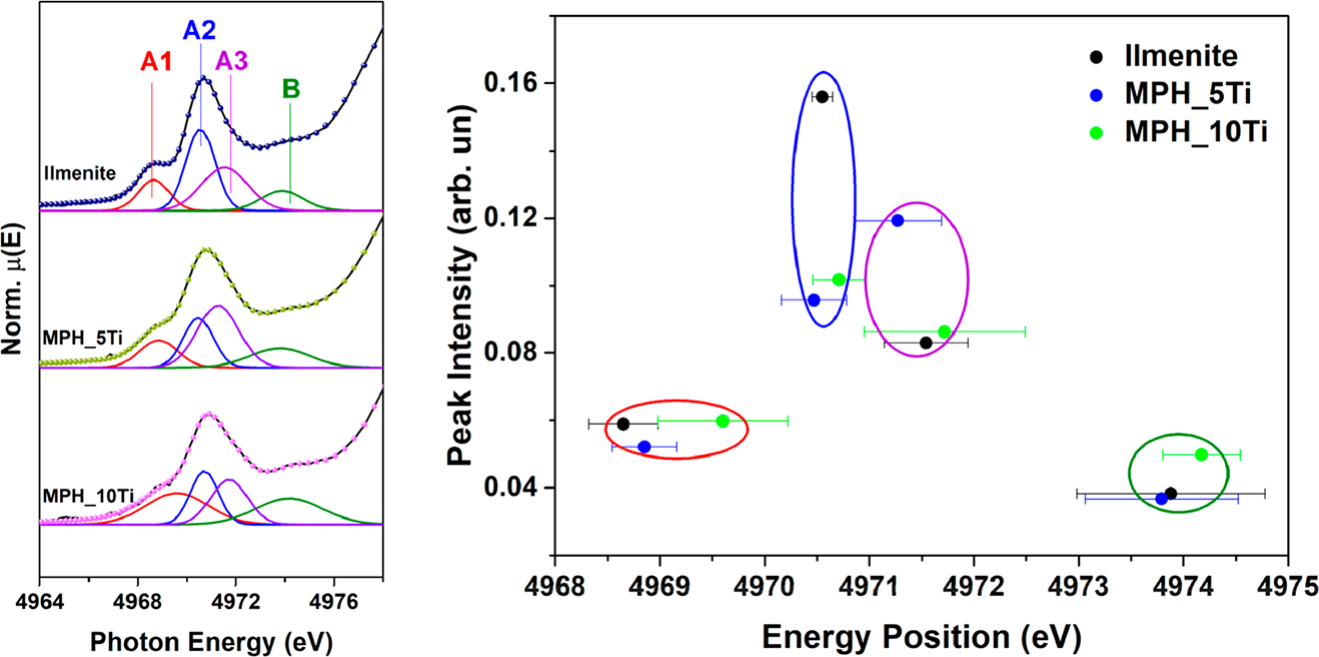
Left: Ti K pre-edge spectra and fits with four Gaussian features (red, peak A1; blue, peak A2; violet, peak A3; green, peak B), shown for Ti-modified MPH and for reference ilmenite. Right: Corresponding intensity and energy plot. The error bars are standard errors on the peak positions as calculated by the Fityk package. The circles are guides to the eye that group data points pertaining to a given feature.

The Ti K edge XANES of Ti-modified MPH photoanodes and of pseudobrookite (Fe_2_TiO_5_), anatase, brookite, rutile, and ilmenite reference samples are shown in Figure 2. The pre-edge features arise from dipole and quadrupole transitions to bound electronic states in the bottom of the conduction band originating from Ti 3d/4p and O 2p hybridization,^24^ and their line shape is strongly affected by the oxidation state and local symmetry around Ti.^25,26^ The pre-edge features are highlighted in the left panel of Figure 3. It is known that the A1, A2, and A3 features are due to transitions to t_2g_ states and feature B to transitions to e_g_ states.^27,28^ The rising and main edge region is attributed to the electronic transition of 1s electrons to continuum states. It can be noted that all spectral features of Ti-modified MPH are similar to those of ilmenite (with some extra broadening of the main edge) and are significantly different from those of all titanium oxides and pseudobrookite. This indicates clearly that Ti is found in a local structure very similar to that of ilmenite.

The pre-edge spectral features of the Ti-modified MPH and ilmenite were fitted by Gaussian components, as shown in the left panel of Figure 3. The numerical results are reported in Table S2. Since it is known that the intensity and the energy position of each peak are related to oxidation state and local coordination, we report these quantities in the right panel of Figure 3 as an intensity versus energy position plot. It is clear that, within the uncertainties, the energies of all components are equal, while there are some differences in the intensities and widths. Our conclusion is that the local electronic structure around Ti near the bottom of the conduction band is similar to that in ilmenite. This result points to a similar atomic structure, which was assessed by Ti K edge EXAFS as described below.

The background-subtracted Ti K edge EXAFS spectra are shown in Figure S3. The spectra of Ti-modified MPH have a line shape similar to the ilmenite spectrum. This indicates that the local coordination around Ti is very similar to the ilmenite local structure, as expected. Ilmenite has trigonal crystal structure with space group 167 (i.e., *R*3̅*c*:*H*) as hematite, and this structure was used to fit the spectra.

**Figure 4:**
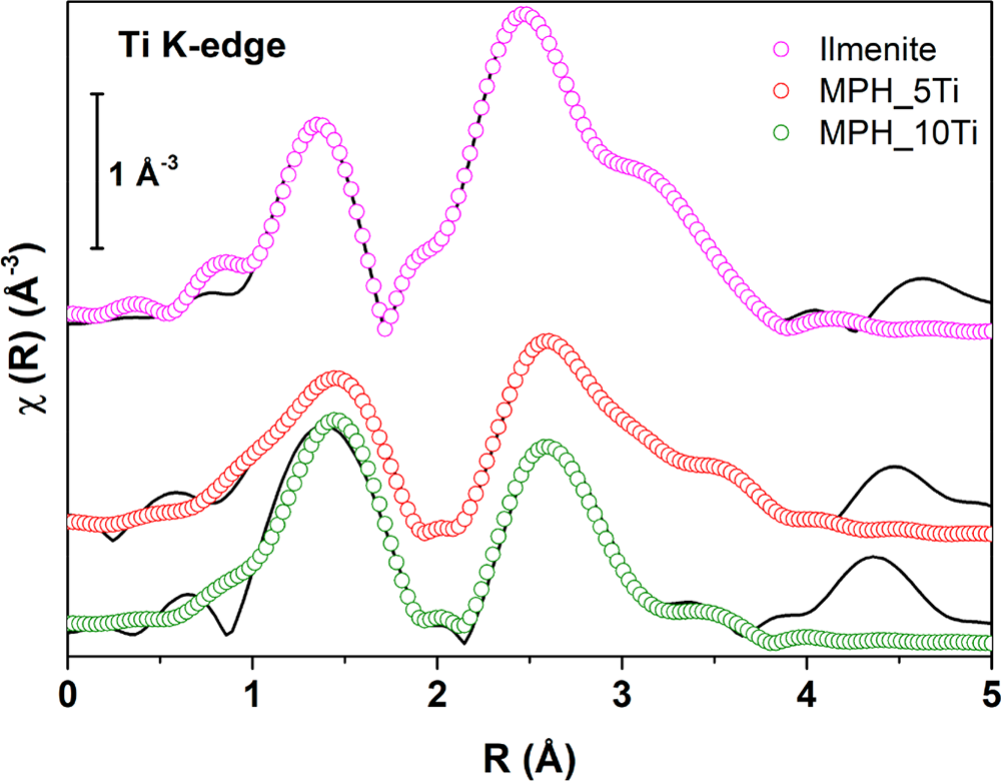
Fourier-transformed Ti K edge EXAFS (*k*^2^-weighted) spectra of Ti-modified MPH and reference ilmenite with their best fits (data reported as continuous line and fits as empty circles). Spectra have been vertically offset for clarity.

The Fourier transforms of Ti K edge EXAFS spectra, obtained in the range of 2.5–9.5 Å^–1^, are shown in Figure 4. The only notable differences between the samples and ilmenite are a small shift to higher distances of the first peak and a smaller intensity of the second peak with respect to the first.

In the quantitative EXAFS analysis, we sought to distinguish between two options: (i) substitutional Ti atoms within the hematite matrix or (ii) local structure around Ti similar to ilmenite. The key difference between the two is the contributions giving rise to the second peak in the Fourier transforms: in the first case only Ti–Fe contributions would be present, while in the second both Ti–Ti and Ti–Fe are expected. Any attempt to fit the samples’ spectra with only Ti–Fe contributions yielded unphysical structural parameters; we thus conclude that the local structure is in fact similar to ilmenite. Therefore, all fits were performed on the basis of the known local structure of ilmenite. The many-body amplitude reduction factor was obtained from the best fit of the ilmenite spectrum (*S*_0_^2^ = 0.84), and this was fixed in the fits of Ti-modified MPH. The other fitting parameters were a common energy origin shift (Δ*E*_0_) and interatomic distances (*R*) and Debye–Waller factors (σ^2^) for each contribution listed below. The first peak in the ilmenite spectrum was fitted with contributions originating from three O atoms at ∼1.84 Å and three more at ∼2.02 Å, the second peak with three Ti atoms at ∼2.99 Å, three Fe atoms at ∼3.41 Å, and six O atoms at ∼3.29 Å, and the third by six Fe atoms at ∼3.76 Å. No multiple scattering contributions were considered due to their low amplitude. The photoanodes spectra were fitted in a similar fashion, and slight variations in the local structural parameters were obtained, as summarized in Table S3. Debye–Waller factors are nearly always higher in Ti-modified MPH, indicating a more structurally distorted environment. A significant difference is that the Ti–O distances in the photoanodes are significantly higher than in ilmenite and tend to the values of the corresponding Fe–O distances in hematite. This might reflect the influence of the hematite matrix on the ilmenite-like regions.

**Figure 5:**
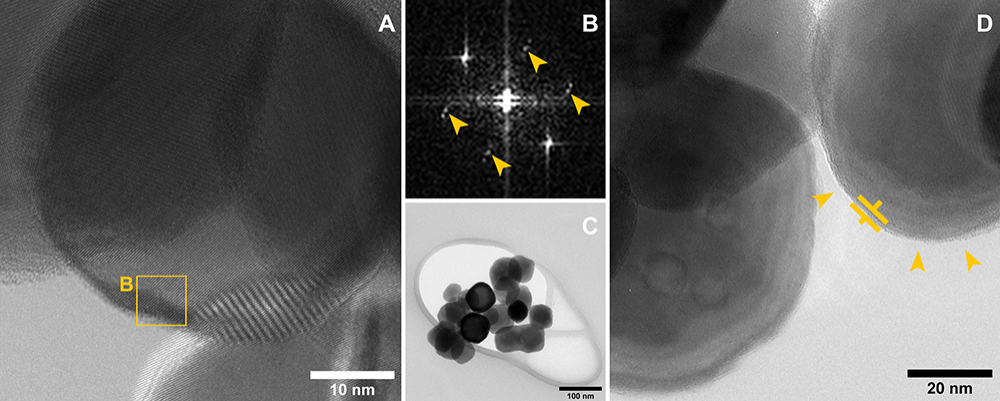
(A) HRTEM micrographs of MPH_5Ti and (B) related fast Fourier transform (FFT) of the selected region of interest; arrows indicate the reflections indexed as ilmenite (104) planes. (C) Low-magnification TEM micrograph and (D) HRTEM micrograph that shows the 2–4 nm thin conformal layer around nanoparticles.

**Figure 6:**
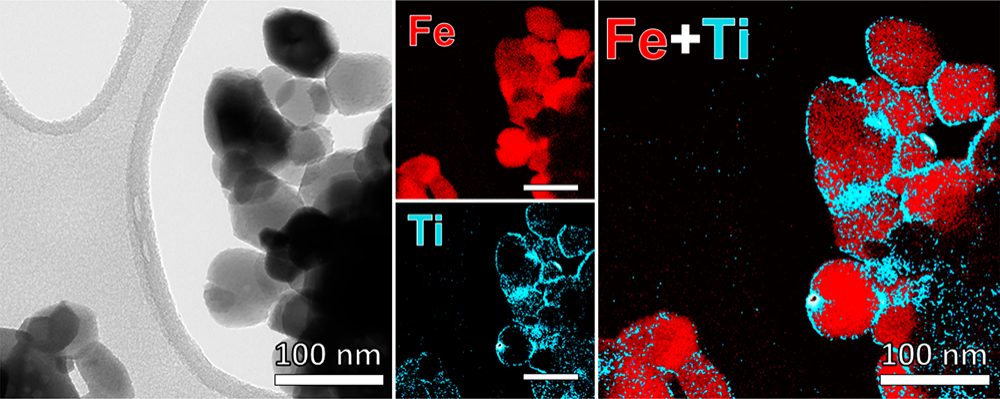
TEM micrographs of MPH_5Ti, related ESI Fe and Ti maps, and the combined map.

This view is reinforced by TEM analyses that additionally provide local information on the phase distribution at the nanoscale. Structural analyses by selected area electron diffraction (SAED) and HRTEM confirm hematite as the main phase but also reveal the presence of ilmenite. SAED analysis (Figure S4) highlights a polycrystalline structure; the diffraction rings characteristic of hematite are clearly visible. Ilmenite shares the trigonal crystal structure with hematite, and despite the fact that part of the reflections partially overlap, some planes specific to ilmenite, such as (101) and (003), are clearly detected in the diffraction micrograph. Moreover, HRTEM structural analysis in proximity of the surface (Figure 5, parts A and B) reveals again the hematite main phase (Figure 5B, zone axis [2 −3 2 ]) together with reflections indexed to ilmenite (104) planes (marked by arrows Figure 5B). From the morphological point of view, the MPH_5Ti sample appears to be constituted by well-shaped crystalline nanoparticles of about 40–70 nm diameter enveloped by a conformal layer with a thickness in the range of 2–4 nm (pointed by arrows in Figure 5, parts C and D). The ESI analysis shown in Figure 6, performed by mapping the Ti and Fe L_2,3_ energy loss feature, clearly shows Ti enrichment of this layer.

**Figure 7:**
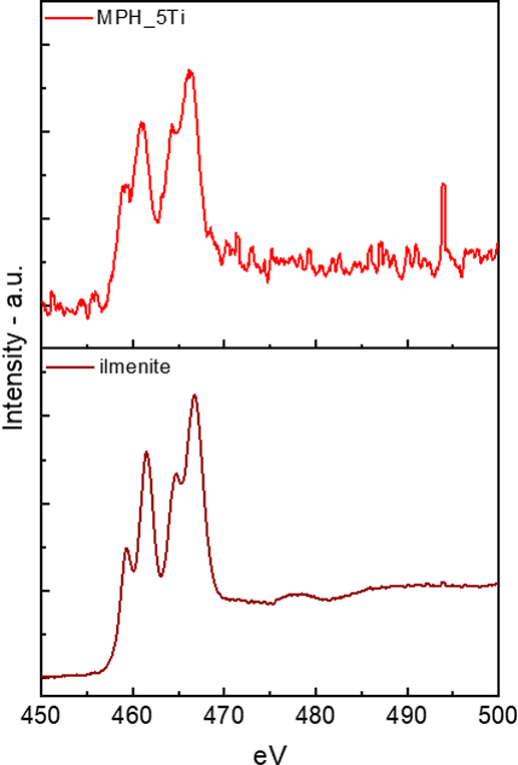
EELS spectra for MPH_5Ti specimen (above) and reference ilmenite (below) recorded under the same experimental conditions at the Ti L_2,3_ edge.

The similarity between the Ti L_2,3_ edge EELS spectra for sample MPH_5Ti and for an ilmenite reference sample (Figure 7) clearly indicates that the Ti-rich outer layers around the nanoparticles are ilmenite, in agreement with the outcome of EXAFS analysis. In fact, the energies of the edge onsets for both the L_2_ edge at 464.3 eV and the L_3_ edge at 458.9 match well the reference ones at 464.7 and 459.3 eV, respectively; moreover, the overall line shape (including the crystal field splitting of each edge and the corresponding branching ratios) of the MPH_5Ti spectrum corresponds quite well with that of the ilmenite reference. Moreover, the EELS spectrum of sample MPH_5Ti differs significantly from those of rutile, anatase, and amorphous TiO_2_ (Figure S5).

Although ilmenite is reported to have a valence band edge which is not properly aligned to accept holes from the valence band (VB) of hematite,^29^ in our case the very thin (2–4 nm) FeTiO_3_ layer does not hinder the hole transfer to the electrolyte, as clearly demonstrated by the significant improvement of the PEC performance of the MPH_5Ti electrodes compared to the MPH counterpart (Figure S1). A favorable band alignment is instead obtained in other kind of composites, such as those constituted by hematite and pseudobrookite Fe_2_TiO_5_.^7,30,31^ In this case, the Fe_2_TiO_5_ phase allows for the formation of a type II heterojunction with α-Fe_2_O_3_, leading to maximum photocurrent values up to 3 mA/cm^2^ when a nickel/iron oxide OEC is introduced.^31^

Furthermore, to the best of our knowledge, our report is the first in which a thoroughly characterized Fe_2_O_3_/FeTiO_3_ composite is used in a photoanodic setup for water oxidation. Indeed, this combination is generally used for its magnetic properties,^32,33^ while photoelectrochemical or photocatalytic applications often exploit FeTiO_3_/TiO_2_ junctions.^34−36^

### 3.2 TAS and TPC Measurements on the Photoanodes

In order to evaluate the effect of the ilmenite phase on the interfacial dynamics of the photogenerated charges, we have performed TAS and TPC measurements on unmodified MPH and on MPH_5Ti, i.e., the best-performing photoanode in terms of Ti(IV) content. The latter was also functionalized with FeOEC with the aim to investigate its role in photoelectrochemical water oxidation.

**Figure 8:**
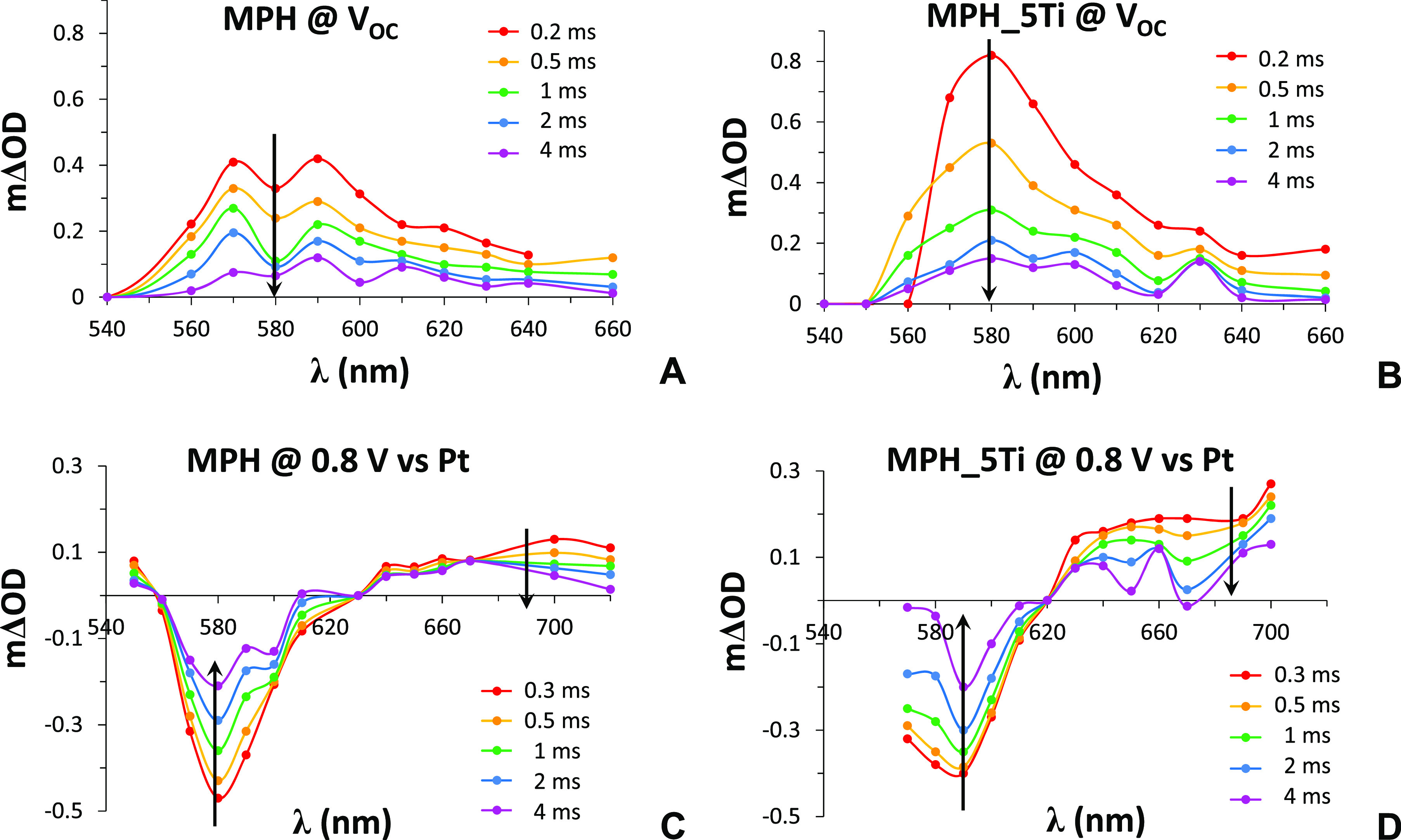
Transient absorption spectra of MPH and MPH_5Ti photoanodes at both *V*_OC_ (A and B) and 0.8 V vs Pt applied bias (C and D).

The TA spectra for MPH and MPH_5Ti photoanodes at *V*_OC_ are reported in Figure 8, parts A and B. The laser excitation power was kept as low as possible in order to observe dynamics that could be related to the behavior of the photoanode under solar illumination, resulting in a small optical density difference (ΔOD) in the order of 10^–4^. TA spectra at *V*_OC_ are dominated by a broad absorption band at wavelengths between 540 and 640 nm peaking around 580–590 nm, which decays in the millisecond time scale. This feature, common to both MPH and MPH_5Ti, can be attributed to photohole trapping in intragap states located a few hundreds of millielectronvolts below the conduction band, as demonstrated by the Durrant group for other nanostructured hematite photoanodes.^37^ In the absence of an applied bias, these intragap states (below the Fermi level) are occupied by electrons and therefore do not contribute to ground-state absorption. After laser pulse excitation, valence band photoholes are rapidly (≪milliseconds) trapped at these states, making them available for optical transitions of valence band electrons that lead to the observed transient absorption band. Its decay is due to the filling of the intragap states by photoexcited electrons in the conduction band. In other words, the intragap states act as recombination centers. The fact that the absorption band is more intense (almost double) in the case of MPH_5Ti points to a higher number of available states for the holes to be trapped in, due to the formation of the hematite–ilmenite heterointerface.

When the photoanodes are biased at 0.8 V versus Pt (the potential at which the limiting photocurrent is observed in a two-electrode configuration; see Figure S1B for the corresponding *J*–*V* curves), the spectral characteristics change. An intense bleach appears at a similar wavelength of *V*_OC_ transient absorption (centered at 580 and 590 nm for MPH and MPH_5Ti, respectively; see Figure 8, parts C and D). Moreover, a broad absorption band at λ > 630 nm extending into the red is visible. This spectral behavior is consistent with previous observations.^37^

The origin of the bleaching can be understood considering again the intragap states, as follows. Under applied anodic bias, optical absorption at 580–590 nm takes place in the ground state because the intragap states are empty, at least within the space charge region. However, after laser excitation, the intragap states are rapidly (≪milliseconds) filled by photoexcited electrons in the conduction band. This reduces absorption at 580–590 nm, generating a negative ΔOD signal, approximately 90% of which is recovered within 10 ms (Figure S6A). Moreover, as reported in Figure S6B, for both MPH and MPH_5Ti electrodes, the bleach intensity increases (in absolute value) with the square root of the applied bias, further confirming that this dynamics originates from the extension of the space charge layer in the photoanode upon positive polarization.

On the other hand, the broad feature at λ > 630 nm has been attributed to photoholes swept by the electric field at the semiconductor/electrolyte interface (SEI).^37^ It is interesting to note that it is at least 2 times higher in the Ti-modified electrode, consistent with the improved photoelectrochemical performance.

**Figure 9:**
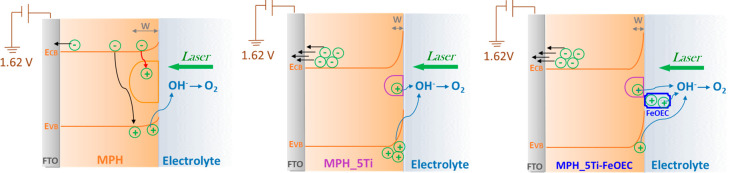
Pictorial representation of the processes occurring at the SEI interfaces of the three different photoanodes under 1.62 V applied bias. The black arrows indicate the processes related to the fast recombination channels competing with electron collection at the back contact, while the red ones indicate slow recombination pathways involving the holes trapped in intragap states. Blue arrows indicate the hole transfers responsible for the interfacial oxygen evolution reaction.

In order to investigate the role of the different components of the photoanodic interfaces in more detail, we have performed TPC measurements. The photocurrent decay was recorded when striking the photoanode from the “front” (i.e., the electrolyte side, as opposed to the FTO collector “back” side) under variable bias (Figure S7). These experiments are schematized in Figure 9, considering the different behavior of the three interfaces under 1.62 V (vs RHE) applied bias and highlighting the possible pathways for the fate of the charge carriers generated by the laser pulse.

**Figure 10:**
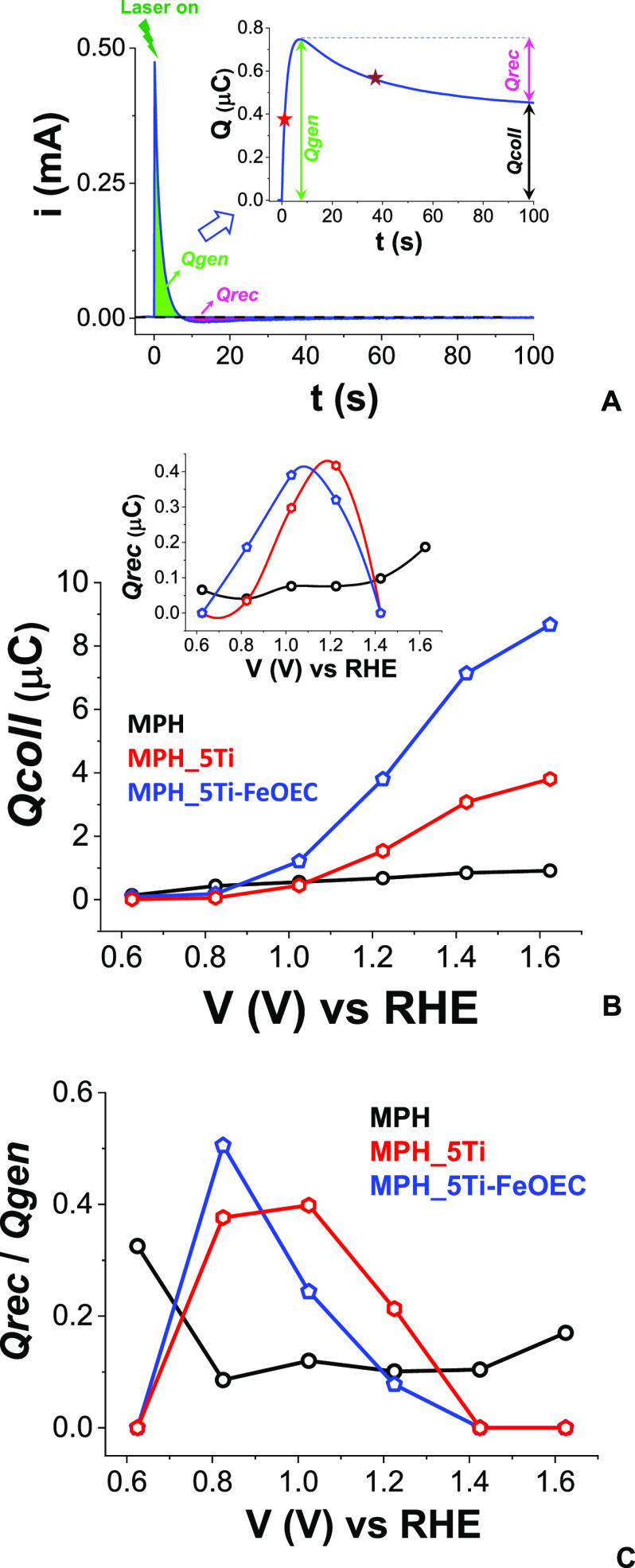
(A) Typical TPC decay and corresponding photogenerated charge trace (inset) obtained after its integration over time, illustrating the contributions due to Qgen, Qrec, and Qcoll. The values indicated with the red and purple stars correspond to the times at half-amplitude τ_1_ and τ_2_ (*vide infra*). In this specific case, the *i*–*t* trace corresponds to a MPH_5Ti photoanode held at 1.02 V bias. (B and C) Applied bias dependence of Qcoll (B) and Qrec (B, inset) and of Qrec/Qgen (C) for MPH, MPH_5Ti, and MPH_5Ti-FeOEC.

Figure 10A reports a typical transient chronoamperometric trace in the 1–100 ms time domain, showing the following distinctive features: (i) a positive photocurrent peak, which is originated by collection of laser-generated electrons at the back contact and usually decays on a 10 ms time scale, in agreement with the transient lifetime observed by TAS. The corresponding photocharge peak rise; in the following we call τ_1_ the time at half-amplitude; (ii) a negative transient (with half-amplitude time as τ_2_), corresponding to a photocharge that extends up to 100 ms, originated by the recombination of the photogenerated electrons with holes trapped in surface states, and thus not injected into the electrolyte. The integral of these transient features yields the temporal evolution of the photogenerated charge (inset of Figure 10A), thus allowing for the identification and quantification of the different processes framed by the two different time regimes (10 and 100 ms). In particular, we define (i) Qgen, as the area subtended by the positive photocurrent peak, which is the result of the kinetic competition between electron transport at the collector and fast recombination processes involving reaction with mobile holes (VB holes) or holes contained in shallow traps, (ii) Qrec, as the charge lost due to slower recombination processes involving long-lived surface-trapped holes, and (iii) Qcoll, as the actual collected charge, given by the difference between the other two contributions, according to
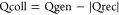
2

We calculate these charges for all the TPC; traces reported in Figure S7. Figure 10B shows the resulting bias dependence of Qcoll and Qrec (inset) for all the investigated photoanodes, while the curves corresponding to Qgen are reported in Figure S8A. In general, the magnitude of the photocurrent transients increases in the order MPH < MPH_5Ti < MPH_5Ti-FeOEC (Figure S7), and the resulting trend in Qcoll is consistent with the *J*–*V* curves obtained under 1 sun illumination (see Figure S1A), confirming that the presence of the ilmenite overlayer improves the collection of photogenerated charge. We note that the Qcoll versus *V* trend reported in Figure 10B is exacerbated compared with the conventional *J*–*V* curves (Figure S1), due to the front-side illumination geometry herein adopted, which is the most unfavorable for the collection of charge carriers in hematite.

Furthermore, the time at which the Qgen reaches half of its peak value (τ_1_; see also inset of Figure 10A) is essentially constant within the explored potential range, being ca. 0.5 ms for MPH, ca. 1.5 ms for MPH_5Ti, and ca. 4 ms for MPH_5Ti-FeOEC (see Figure S8B). This suggests that fast recombination processes are partially inhibited by the presence of the ilmenite layer, allowing it to extend the useful time frame for the collection of photogenerated electrons.

The applied bias dependence of Qrec (inset of Figure 10B) gives further insight on the differences in the photoanodic performance. Indeed, we obtain a distribution of low (ca. 0.05 μC) and almost constant values for MPH, meaning that the fraction of holes that are able to survive fast recombination and undergo slow recombination with electrons is smaller in such electrodes. On the contrary, a well-defined bell-shaped behavior is observed for both MPH_5Ti and MPH_5Ti-FeOEC within the interval 0.6 ≤ *V* ≤ 1.4 V, indicating that a more significant fraction of holes is now trapped in long-lived surface states, as a result of the interfacial modification. The distribution of Qrec peaks between 1.0 and 1.2 V, in agreement with the surface chemical capacitance distribution found by EIS experiments in these (*vide infra*) and other types of bare and Ti-modified hematite electrodes.^7,13,17,38,39^ We note that the Qrec/Qgen ratio, shown in the inset of Figure 10C, is higher in the Ti-modified electrodes and that, contrary to MPH, it drops to 0 for an applied potential ≥1.4 V. This behavior can be explained by the buildup of a stronger electric field inside the Ti-modified electrodes compared to unmodified ones as the positive potential increases. The enhanced field sweeps electrons away from the surface to the collector, leaving holes free to react with water. We can argue that the space charge inside unmodified hematite does not develop enough to guarantee efficient collection, while the structural changes induced by Ti incorporation at the interface allow it to sustain a stronger electric field and bring about a redistribution of surface states. These effects, pointed out by the Andreu group for Fe_2_O_3_/Fe_2_TiO_5_ heterojunctions,^7^ may be also related to Fermi-level unpinning that was observed in the presence of a thin buffer layer of hematite underneath the MPH and greatly improved electron collection.^17^ It is worth noting that, in the case of MPH_5Ti-FeOEC, hole trapping in the catalyst’s states causes the formation of high-valence iron species,^40^ which are in turn able to oxidize water and evolve oxygen.

Cyclic voltammetry (CV) in the dark can be useful to investigate the role and the nature of the donor states in Ti-modified hematite and to verify the hypothesis that the ilmenite overlayer passivates surface states. The CV curves reported in Figure S9A were recorded after preconditioning at 1.6 V, in order to extract charge from donor states close to the conduction band edge. The potential was then scanned from 1.6 V to the cathodic direction, until the threshold of the conduction band edge of the semiconductor was reached, and then backward to the initial potential. We observe in both MPH and MPH_5Ti a well-defined wave, which is more reversible and intense (about a factor 4.5) in the latter sample, centered around 0 V and originating from the filling of donor states (*N*_D_) close to the semiconductor band edge.^41^ A similar response was recorded for MPH_5Ti-FeOEC. The increased *N*_D_ value is consistent with a Mott–Schottky (MS) analysis carried out on similar Ti(IV)-modified electrodes, where a 3-fold increase in *N*_D_ was observed.^13^ Herein, however, we have preferred to explore the CV response, rather than perform high-frequency electrochemical impedance spectroscopy, due both to difficulties in knowing the dielectric constant of the modified interface and to the issue of frequency dispersion,^42^ which makes the evaluation of the donor density by MS analysis quite uncertain. Prior to the main cathodic wave, unmodified MPH also shows a broad prewave at ca. 0.7 V. This feature, well-evident when the voltammetric curves are compared at the same current density (Figure S9B), is absent in the Ti-modified samples. The prewave can be ascribed to the presence of deep electron traps at about 400 meV below the conduction band (CB) edge,^43^ which may pin the Fermi level of MPH and cause recombination. Its absence in Ti-modified MPH indicates that the ilmenite overlayer acts as a deep trap passivating agent, while also inducing a greater number of oxygen vacancies, which were reported to improve the photocurrent response of hematite through the reduction of bulk recombination. This performance was attributed both to an increase in film conductivity and to a stronger depletion field, supported by the higher donor density.^44,45^

**Figure 11:**
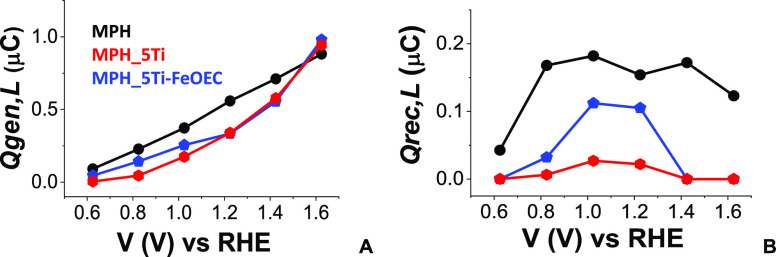
Applied bias dependence of Qgen,L (A) and Qrec,L (B) in the presence of a strong white light stimulus provided by a solar simulator shining from the back contact.

**Figure 12:**
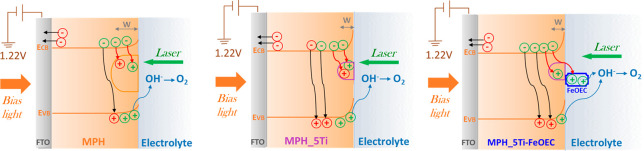
Pictorial representation of the three different interfaces in the presence of an additional white light illumination and under 1.22 V applied bias. Laser-generated carriers are indicated in green, while the steady-state populations of electrons and holes are in red. The black arrows indicate the processes related to Qgen,L, while the red ones indicate recombination pathways involving intragap states (Qrec,L). The blue arrows indicate the hole transfers responsible for the interfacial reaction of oxygen evolution.

With the aim to unravel space charge effects, we also carried out TPC experiments under additional white light illumination provided by a solar simulator set to 0.4 W/cm^2^ irradiance (see Figure S10 for a typical *J*–*V* curve obtained under these conditions). This condition creates a large steady-state population of charge carriers, which is slightly perturbed by the laser pulse. Under such conditions, the generated and recombined charges are termed Qgen,L and Qrec,L; their dependence on the applied bias is reported in Figure 11. In Figure 12, we report a schematic representation of the charge-transfer dynamics when both the laser and the white light sources shine from opposite directions under 1.22 V applied potential. This intermediate bias was chosen as a representative voltage at which the various dynamics occurring at different time scales can be simultaneously observed.

Strikingly, under white light illumination the magnitude of the photocurrent transients in the best-performing Ti-modified MPH electrodes is strongly reduced, resulting in Qgen,L values that are ca. 5 and 10 times lower than Qgen for MPH_5Ti and MPH_5Ti-FeOEC, respectively. By contrast, Qgen,L remains substantially unvaried with respect to the dark counterpart in the case of MPH. Figure 11A shows that Qgen,L values are higher (up to 1.4 V) in unmodified MPH compared to Ti-modified MPH, in spite of its poorer photoanodic performance. At the same time, Qgen,L maintains the expected increase when the bias becomes more anodic. This counterintuitive behavior can be rationalized in terms of charge recombination at the surface of the electrodes between electrons produced by the laser pulse and holes generated by white light. It should be recalled that the laser pulse strikes from the electrolyte side and that the penetration depth of 355 nm radiation in MPH films is estimated to be in the 25–50 nm range [note: the optical penetration depth (α) of 355 nm photons in our hematite samples was directly calculated from the film thickness (*l*) and absorbance (*A*), using the formula α (cm^–1^) = ln(10)*A*/*l* (cm)], in good agreement with the known extinction coefficient of hematite.^46^ The constant white light illumination generates electrons and holes, which are swept in opposite directions by the electric field created inside the photoanode. Electrons travel to the collector, while holes accumulate at the SEI. The stronger the electric field at a given potential, the higher the hole density at the interface with the electrolyte. Thus, in the electrodes displaying a stronger depletion layer, i.e., MPH_5Ti and MPH_5Ti-FeOEC, the laser pulse generates electrons in a spatial region densely populated by holes, causing fast recombination and decreasing the intensity of Qgen and Qcoll transients. In agreement with this interpretation, the time at half-amplitude measured for Qgen,L (named τ_1,L_ and reported in Figure S11) is ca. half of the dark τ_1_ value for MPH_5Ti and up to 4 times lower than τ_1_ for MPH_5Ti-FeOEC (compare Figures S8B and S11). On the contrary, τ_1,L_ and τ_1_ are very similar in the case of MPH photoanodes. The stronger decrease in the τ_1,L_ values in the presence of the catalyst can be well-explained by the occupation of the surface FeOEC sites by the photogenerated holes at *V* ≥ 1.0 V.

Due to fast recombination within the penetration depth of 355 nm laser pulses, Qrec,L also decreases by at least a factor of 5 with respect to Qrec for both MPH_5Ti and MPH_5Ti-FeOEC (compare Figures 11B and 10B). In any case, Qrec,L maintains the bell-shaped distribution observed in the absence of white light bias and still drops to 0 for *V* ≥ 1.4 V. After this threshold, a relative increase in both Qgen,L and, as a consequence, Qcoll,L can be also observed (Figure S12). Furthermore, the MPH_5Ti-FeOEC photoanode displays higher Qrec,L values with respect to those of MPH_5Ti. This finding suggests that, in the presence of the amorphous and electrolyte-permeable catalyst, an increased number of electronic states are available on the FeOEC for the localization of photogenerated holes.^16,38^

The transient photocharge dynamics of MPH in the presence of white light illumination are consistent with the presence of a thinner space charge layer, which can be ascribed to Fermi-level pinning by deep traps, as previously discussed. Indeed, for MPH the Qrec,L values are almost constant for *V* ≥ 0.8 V and approximately doubled with respect to dark Qrec values. A plausible interpretation is that holes generated under white light and trapped in the MPH surface states do not experience a significant surface accumulation upon application of anodic bias. The larger number of photoproduced holes, with respect to the dark case, enhances their probability of slow recombination with the laser-produced electrons, explaining the increase in Qrec,L.

**Figure 13:**
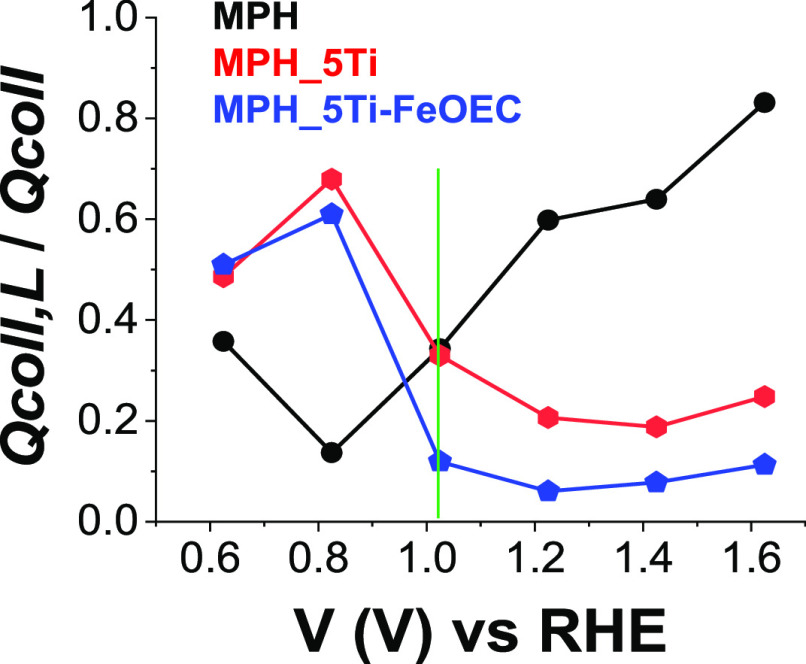
Applied bias dependence of Qcoll,L/Qcoll.

The overall behavior of the analyzed photoanodes is summarized in Figure 13, in which we report the bias dependence of the ratio between the transiently collected charge in the presence (Qcoll,L) and in the absence (Qcoll) of the white light source. Once again, the observed trends clearly illustrate the differences between MPH and the ilmenite-modified MPH photoelectrodes in terms of charge separation. When the FeTiO_3_ phase at the interface with the electrolyte is present, the Qcoll,L/Qcoll drops as soon as the voltage reaches 1.0 V due to accumulation of photoholes at the SEI where laser-induced electron generation occurs. These surface hole localization effects are strongest in the best-performing electrode (MPH_5Ti-FeOEC), in which the steepest decrease to the lowest Qcoll,L/Qcoll is related to the highest photocurrent and clearly tied to photocurrent generation, as can be appreciated from the onset of the *J*–*V* characteristics between 0.92 and 1.02 V (Figure S1A). Thus, although the band edges of ilmenite are not properly aligned with those of hematite to promote a downhill electron-transfer cascade capable to enhance charge separation, they contribute to heterointerfacial effects, which equally result in the suppression of carrier recombination. Hence, by combining the information gained from both TPC and CV experiments, we can conclude that the formation of the FeTiO_3_ overlayer passivates deep traps, which otherwise cause recombination, and induce a greater concentration of donor levels close to the conduction band edge, building up a stronger depletion layer.

The insights gained by TPC measurements were corroborated by means of EIS under illumination. The resulting Nyquist plots are reported in Figure S13A–C and fitted using the equivalent circuit model indicated in Figure S13D. The elements of this particular circuit, often used to model hematite-based photoanodes,^38,47,48^ include the space charge capacitance of hematite (*C*_SC_), the charge-transport resistance through the space charge (*R*_SC_), the surface-state capacitance (*C*_SS_), and the resistance of the charge transfer from the surface states to the electrolyte (*R*_CT,SS_). While *C*_SC_ and *R*_SC_ are related to high-frequency (i.e., faster) processes, *R*_CT,SS_ and *C*_SS_ are associated with the low-frequency (slower) processes taking place at the interface with the electrolyte. This results in Nyquist plots composed by two different arcs, which in our specific case collapse into a single one at high applied bias (*V* ≥ 1.4 V).

The adequacy of the circuit model is confirmed by the good correlation observed when the plots of the differential resistances (d*I*/d*V*, calculated from the *I*–*V* curves) versus the applied potential are compared to the corresponding *R*_TOT_^–1^ values (Figure S14). In particular, the total resistance (*R*_TOT_) is calculated by adding *R*_SC_, *R*_CT,SS_, and the serial resistance (*R*_S_).

The EIS data also confirmed the expected bell-shaped distribution of *C*_SS_ for the Ti-modified electrodes (Figure S15A), exhibiting at least 1 order of magnitude higher values with respect to those of MPH. This observation is consistent with the better charge separation observed for MPH_5Ti and MPH_5Ti-FeOEC electrodes, yielding a higher surface concentration of high-valence iron–oxo species promoting water oxidation.^39^ As a result, the corresponding *R*_CT,SS_ values are considerably lower with respect to those of MPH (Figure S15B). A further contribution to the higher *C*_SS_ values can be ascribed to the presence of a larger surface concentration of Fe(III)–OH upon Ti incorporation, as reported by the Hamann group for atomic layer deposited hematite electrodes.^49^

Moreover, when MPH_5Ti is functionalized with FeOEC, the *C*_SS_ values further increase (up to a factor 4 with respect to MPH_5Ti) and the differential capacitance peak shifts slightly in the cathodic direction. This confirms the picture of hole trapping into catalyst sites during MPH_5Ti-FeOEC operation. At the same time, it is worth noting that the maximum *C*_SS_ nicely correlates with both the inflection point of the *J*–*V* curve and the minimum value of *R*_CT,SS_ (observed at 1.22 V for MPH_5Ti-FeOEC, see Figure S16).

Finally, we used the EIS data to calculate the efficiency for the water oxidation process (η_WO_), as reported by the groups of Peter^50^ and Mendes^51^ and briefly illustrated in the Supporting Information.

**Figure 14:**
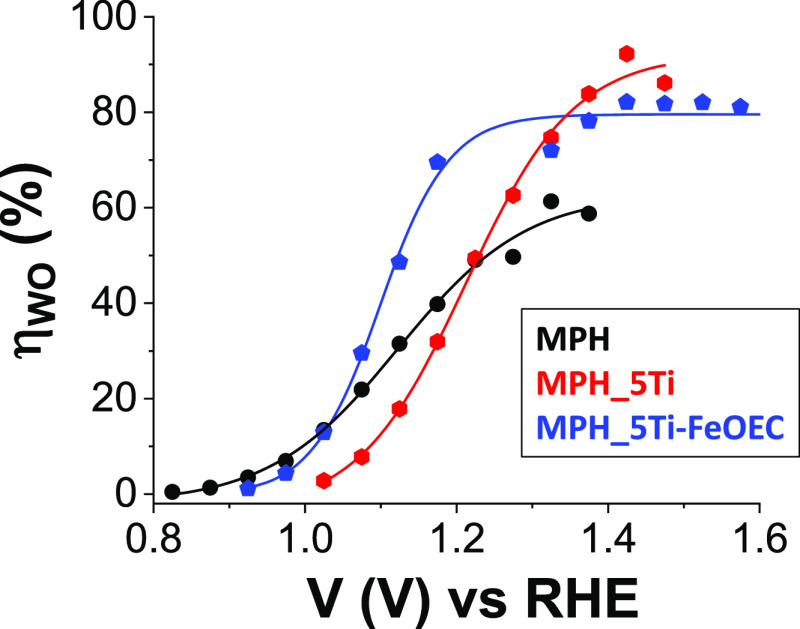
Applied bias dependence of the efficiency of water oxidation (η_WO_) for MPH (black), MPH_5Ti (red), and MPH_5Ti-FeOEC (blue) photoanodes recorded in 0.1 M KOH (pH 13.3) under 1 sun (0.1 W/cm^2^, AM1.5G) illumination.

Figure 14 reports the η_WO_ values obtained for the different photoanodes, which, as expected, mirror the trend of the *J*–*V* curves (see also Figure S1A). Indeed, the η_WO_ accounts for the fact that only the photogenerated charges able to survive recombination can reach the interface and perform water oxidation. The best results were obtained for MPH_5Ti-FeOEC electrodes that are able to operate with 80% efficiency already at applied potentials close to the thermodynamic value for water oxidation. These results are in line with those obtained for other hematite-based interfaces functionalized with different metal oxides catalysts, e.g., iridium/ruthenium-based OECs^51^ and nickel/iron-based OECs.^48^

## 4 Conclusions

State-of-the-art XANES and EXAFS measurements have been used to gain advanced structural/chemical knowledge about the origin of the enhanced PEC performance in Ti(IV)-modified mesoporous hematite photoanodes. In particular, we demonstrate that the inclusion of Ti(IV) does not influence the local structure of Fe, which remains that of hematite, and that the local electronic and atomic structures around Ti are similar to those of ilmenite (FeTiO_3_).

As illustrated by TAS, TPC, and EIS analyses, the formation of this mixed phase results in a main beneficial mechanistic pathway for the modified interfaces, related to the passivation of deep electron traps, which act as recombination centers and cause Fermi-level pinning in unmodified hematite. At the same time, it induces a higher concentration of oxygen vacancies acting as donor states close to the conduction band edge. Therefore, better charge separation is possible thanks to the buildup of a stronger electric field inside the semiconductor, which directs holes at the interface with the electrolyte.

Despite the misalignment between hematite and ilmenite band edges, the thin and discontinuous nature of the FeTiO_3_ layer allows for a sufficient coupling between the surface-trapped holes and water for the charge transfer to occur.

We also confirmed that the functionalization of the Fe_2_O_3_/FeTiO_3_ interface with an electrolyte-permeable and amorphous catalyst (FeOEC) yields a ternary composite electrode with enhanced hole injection capabilities toward the electrolyte, resulting in photocurrents up to 1.45 mA/cm^2^ at 1.7 V versus RHE.

The present understanding of charge-transfer dynamics in low-cost and highly stable materials, made of earth-abundant and nontoxic elements, will guide the development of tailored composite photoanodes for solar water splitting with enhanced photoelectrochemical outputs.

## Supporting Information Available

Photoanodes preparation, experimental details for transient measurements and EIS, *J*–*V* curves, EXAFS spectra and tables of results, selected area electron diffraction, EELS spectra, transient absorption spectroscopy, transient photocurrent traces and data analyses, cyclic voltammetry, electrochemical impedance spectroscopy (Nyquist plots and circuit-based analyses) (PDF)

## References

[ref1] Lewis N. S. Research opportunities to advance solar energy utilization. Science 2016, 351, aad1920 10.1126/science.aad1920. 26798020

[ref2] Montoya J. H.; Seitz L. C.; Chakthranont P.; Vojvodic A.; Jaramillo T. F.; Nørskov J. K. Materials for solar fuels and chemicals. Nat. Mater. 2017, 16 (1), 70–81. 10.1038/nmat4778. 27994241

[ref3] Walter M. G.; Warren E. L.; McKone J. R.; Boettcher S. W.; Mi Q.; Santori E. A.; Lewis N. S. Solar water splitting cells. Chem. Rev. 2010, 110 (11), 6446–6473. 10.1021/cr1002326. 21062097

[ref4] Tachibana Y.; Vayssieres L.; Durrant J. R. Artificial photosynthesis for solar water-splitting. Nat. Photonics 2012, 6 (8), 511 10.1038/nphoton.2012.175.

[ref5] Sivula K.; Le Formal F.; Grätzel M. Solar water splitting: progress using hematite (α-Fe_2_O_3_) photoelectrodes. ChemSusChem 2011, 4 (4), 432–449. 10.1002/cssc.201000416. 21416621

[ref6] Sharma P.; Jang J. W.; Lee J. S. Key Strategies to Advance the Photoelectrochemical Water Splitting Performance of α-Fe_2_O_3_ Photoanode. ChemCatChem 2019, 11 (1), 157–179. 10.1002/cctc.201801187.

[ref7] Monllor-Satoca D.; Bärtsch M.; Fabrega C.; Genç A.; Reinhard S.; Andreu T.; Arbiol J.; Niederberger M.; Morante J. R. What do you do, titanium? Insight into the role of titanium oxide as a water oxidation promoter in hematite-based photoanodes. Energy Environ. Sci. 2015, 8 (11), 3242–3254. 10.1039/C5EE01679G.

[ref8] Yang X.; Liu R.; Du C.; Dai P.; Zheng Z.; Wang D. Improving hematite-based photoelectrochemical water splitting with ultrathin TiO_2_ by atomic layer deposition. ACS Appl. Mater. Interfaces 2014, 6 (15), 12005–12011. 10.1021/am500948t. 25069041

[ref9] Boscherini F. X-ray absorption fine structure in the study of semiconductor heterostructures and nanostructures. In Characterization of Semiconductor Heterostructures and Nanostructures; Lamberti C., Ed.; Elsevier: Amsterdam, Netherlands, 2008; pp 289–330.

[ref10] Synchrotron Radiation; Mobilio S., Boscherini F., Meneghini C., Eds.; Springer: Heidelberg, Germany, 2016.

[ref11] X-ray Absorption Spectroscopy of Semiconductors; Schnohr C. S., Ridgway M. C., Eds.; Springer: Heidelberg, Germany, 2015.

[ref12] Tang P.; Arbiol J. Engineering surface states of hematite based photoanodes for boosting photoelectrochemical water splitting. Nanoscale Horiz. 2019, 4 (6), 1256–1276. 10.1039/C9NH00368A.

[ref13] Dalle Carbonare N.; Boaretto R.; Caramori S.; Argazzi R.; Dal Colle M.; Pasquini L.; Bertoncello R.; Marelli M.; Evangelisti C.; Bignozzi C. A. Photoelectrochemical Behavior of Electrophoretically Deposited Hematite Thin Films Modified with Ti (IV). Molecules 2016, 21 (7), 942 10.3390/molecules21070942. PMC6273019 27447604

[ref14] Zong X.; Thaweesak S.; Xu H.; Xing Z.; Zou J.; Lu G. M.; Wang L. A scalable colloidal approach to prepare hematite films for efficient solar water splitting. Phys. Chem. Chem. Phys. 2013, 15 (29), 12314–12321. 10.1039/c3cp52153b. 23778329

[ref15] Cristino V.; Berardi S.; Caramori S.; Argazzi R.; Carli S.; Meda L.; Tacca A.; Bignozzi C. A. Efficient solar water oxidation using photovoltaic devices functionalized with earth-abundant oxygen evolving catalysts. Phys. Chem. Chem. Phys. 2013, 15 (31), 13083–13092. 10.1039/c3cp52237g. 23820552

[ref16] Dalle Carbonare N.; Cristino V.; Berardi S.; Carli S.; Argazzi R.; Caramori S.; Meda L.; Tacca A.; Bignozzi C. A. Hematite photoanodes modified with an FeIII water oxidation catalyst. ChemPhysChem 2014, 15 (6), 1164–1174. 10.1002/cphc.201301143. 24643917

[ref17] Dalle Carbonare N.; Carli S.; Argazzi R.; Orlandi M.; Bazzanella N.; Miotello A.; Caramori S.; Bignozzi C. A. Improvement of the electron collection efficiency in porous hematite using a thin iron oxide underlayer: towards efficient all-iron based photoelectrodes. Phys. Chem. Chem. Phys. 2015, 17 (44), 29661–29670. 10.1039/C5CP04152J. 26477966

[ref18] Ravel B.; Newville M. ATHENA, ARTEMIS, HEPHAESTUS: data analysis for X-ray absorption spectroscopy using IFEFFIT. J. Synchrotron Radiat. 2005, 12 (4), 537–541. 10.1107/S0909049505012719. 15968136

[ref19] Wojdyr M. Fityk: a general-purpose peak fitting program. J. Appl. Crystallogr. 2010, 43 (5–1), 1126–1128. 10.1107/S0021889810030499.

[ref20] Malara F.; Minguzzi A.; Marelli M.; Morandi S.; Psaro R.; Dal Santo V.; Naldoni A. α-Fe_2_O_3_/NiOOH: an effective heterostructure for photoelectrochemical water oxidation. ACS Catal. 2015, 5 (9), 5292–5300. 10.1021/acscatal.5b01045.

[ref21] Klinger M. More features, more tools, more CrysTBox. J. Appl. Crystallogr. 2017, 50 (4), 1226–1234. 10.1107/S1600576717006793.

[ref22] Wong S.-T.; Lee J.-F.; Cheng S.; Mou C.-Y. In-situ study of MCM-41-supported iron oxide catalysts by XANES and EXAFS. Appl. Catal., A 2000, 198 (1–2), 115–126. 10.1016/S0926-860X(99)00516-5.

[ref23] Blake R.; Hessevick R.; Zoltai T.; Finger L. W. Refinement of the hematite structure. Am. Mineral. 1966, 51 (1–2), 123–129.

[ref24] Chen S.; Huang M.; Lin P.; Jeng H.; Lee J.; Haw S.; Chen S.; Lin H.; Lu K.; Chen D.; et al. Orbital structure of FeTiO_3_ ilmenite investigated with polarization-dependent X-ray absorption spectroscopy and band structure calculations. Appl. Phys. Lett. 2013, 102 (4), 042107 10.1063/1.4789992.

[ref25] Luca V.; Djajanti S.; Howe R. F. Structural and electronic properties of sol– gel titanium oxides studied by X-ray absorption spectroscopy. J. Phys. Chem. B 1998, 102 (52), 10650–10657. 10.1021/jp981644k.

[ref26] Wu Z.; Ouvrard G.; Gressier P.; Natoli C. Ti and OK edges for titanium oxides by multiple scattering calculations: Comparison to XAS and EELS spectra. Phys. Rev. B: Condens. Matter Mater. Phys. 1997, 55 (16), 10382 10.1103/PhysRevB.55.10382.

[ref27] Cabaret D.; Bordage A.; Juhin A.; Arfaoui M.; Gaudry E. First-principles calculations of X-ray absorption spectra at the K-edge of 3d transition metals: an electronic structure analysis of the pre-edge. Phys. Chem. Chem. Phys. 2010, 12 (21), 5619–5633. 10.1039/b926499j. 20431827

[ref28] Henderson G. S.; De Groot F. M.; Moulton B. J. X-ray absorption near-edge structure (XANES) spectroscopy. Rev. Mineral. Geochem. 2014, 78 (1), 75–138. 10.2138/rmg.2014.78.3.

[ref29] Gu D.; Qin Y.; Wen Y.; Qin L.; Seo H. J. Photochemical and magnetic activities of FeTiO_3_ nanoparticles by electro-spinning synthesis. J. Taiwan Inst. Chem. Eng. 2017, 78, 431–437. 10.1016/j.jtice.2017.04.003.

[ref30] Deng J.; Lv X.; Liu J.; Zhang H.; Nie K.; Hong C.; Wang J.; Sun X.; Zhong J.; Lee S.-T. Thin-layer Fe_2_TiO_5_ on hematite for efficient solar water oxidation. ACS Nano 2015, 9 (5), 5348–5356. 10.1021/acsnano.5b01028. 25885275

[ref31] Tang P.; Xie H.; Ros C.; Han L.; Biset-Peiró M.; He Y.; Kramer W.; Rodríguez A. P.; Saucedo E.; Galán-Mascarós J. R.; et al. Enhanced photoelectrochemical water splitting of hematite multilayer nanowire photoanodes by tuning the surface state via bottom-up interfacial engineering. Energy Environ. Sci. 2017, 10 (10), 2124–2136. 10.1039/C7EE01475A.

[ref32] Matoba T.; Fujita K.; Murai S. Structure and Magnetic Properties of Fe2O3-FeTiO3 Films. Journal of Physics: Conference Series, Vol. 200; IOP Publishing: Bristol, U.K., 2010; p 072029 10.1088/1742-6596/200/7/072029.

[ref33] Robinson P.; Harrison R. J.; McEnroe S. A.; Hargraves R. B. Nature and origin of lamellar magnetism in the hematite-ilmenite series. Am. Mineral. 2004, 89 (5–6), 725–747. 10.2138/am-2004-5-607. 12152075

[ref34] Kim Y. J.; Gao B.; Han S. Y.; Jung M. H.; Chakraborty A. K.; Ko T.; Lee C.; Lee W. I. Heterojunction of FeTiO_3_ nanodisc and TiO_2_ nanoparticle for a novel visible light photocatalyst. J. Phys. Chem. C 2009, 113 (44), 19179–19184. 10.1021/jp908874k.

[ref35] Chen J.; Li Z.; Ma Y.; Sun T. Visible Light-driven FeTiO_3_/TiO_2_ Composite Materials for Pollutant Photodegradation. Chem. Lett. 2016, 45 (11), 1319–1320. 10.1246/cl.160652.

[ref36] Wang J.; Xue C.; Yao W.; Liu J.; Gao X.; Zong R.; Yang Z.; Jin W.; Tao D. MOF-derived hollow TiO_2_@C/FeTiO_3_ nanoparticles as photoanodes with enhanced full spectrum light PEC activities. Appl. Catal., B 2019, 250, 369–381. 10.1016/j.apcatb.2019.03.002.

[ref37] Barroso M.; Pendlebury S. R.; Cowan A. J.; Durrant J. R. Charge carrier trapping, recombination and transfer in hematite (α-Fe_2_O_3_) water splitting photoanodes. Chem. Sci. 2013, 4 (7), 2724–2734. 10.1039/c3sc50496d.

[ref38] Orlandi M.; Dalle Carbonare N.; Caramori S.; Bignozzi C. A.; Berardi S.; Mazzi A.; El Koura Z.; Bazzanella N.; Patel N.; Miotello A. Porous versus compact nanosized Fe (III)-based water oxidation catalyst for photoanodes functionalization. ACS Appl. Mater. Interfaces 2016, 8 (31), 20003–20011. 10.1021/acsami.6b05135. 27447454

[ref39] Klahr B.; Gimenez S.; Fabregat-Santiago F.; Bisquert J.; Hamann T. W. Electrochemical and photoelectrochemical investigation of water oxidation with hematite electrodes. Energy Environ. Sci. 2012, 5 (6), 7626–7636. 10.1039/c2ee21414h.

[ref40] Peter L. M.; Wijayantha K. U.; Tahir A. A. Kinetics of light-driven oxygen evolution at α-Fe_2_O_3_ electrodes. Faraday Discuss. 2012, 155, 309–322. 10.1039/C1FD00079A. 22470982

[ref41] de Carvalho V. A.; Luz R. A. d. S.; Lima B. H.; Crespilho F. N.; Leite E. R.; Souza F. L. Highly oriented hematite nanorods arrays for photoelectrochemical water splitting. J. Power Sources 2012, 205, 525–529. 10.1016/j.jpowsour.2012.01.093.

[ref42] Kavan L.; Grätzel M. Highly efficient semiconducting TiO_2_ photoelectrodes prepared by aerosol pyrolysis. Electrochim. Acta 1995, 40 (5), 643–652. 10.1016/0013-4686(95)90400-W.

[ref43] Bisquert J.; Fabregat-Santiago F.; Mora-Seró I.; Garcia-Belmonte G.; Barea E. M.; Palomares E. A review of recent results on electrochemical determination of the density of electronic states of nanostructured metal-oxide semiconductors and organic hole conductors. Inorg. Chim. Acta 2008, 361 (3), 684–698. 10.1016/j.ica.2007.05.032.

[ref44] Pu A.; Deng J.; Li M.; Gao J.; Zhang H.; Hao Y.; Zhong J.; Sun X. Coupling Ti-doping and oxygen vacancies in hematite nanostructures for solar water oxidation with high efficiency. J. Mater. Chem. A 2014, 2 (8), 2491–2497. 10.1039/c3ta14575a.

[ref45] Wang Z.; Mao X.; Chen P.; Xiao M.; Monny S. A.; Wang S.; Konarova M.; Du A.; Wang L. Understanding the roles of oxygen vacancies in hematite-based photoelectrochemical processes. Angew. Chem. 2019, 131 (4), 1042–1046. 10.1002/ange.201810583. 30417505

[ref46] Marusak L. A.; Messier R.; White W. B. Optical absorption spectrum of hematite, αFe_2_O_3_ near IR to UV. J. Phys. Chem. Solids 1980, 41 (9), 981–984. 10.1016/0022-3697(80)90105-5.

[ref47] Klahr B.; Gimenez S.; Fabregat-Santiago F.; Hamann T.; Bisquert J. Water oxidation at hematite photoelectrodes: the role of surface states. J. Am. Chem. Soc. 2012, 134 (9), 4294–4302. 10.1021/ja210755h. 22303953

[ref48] Orlandi M.; Berardi S.; Mazzi A.; Caramori S.; Boaretto R.; Nart F.; Bignozzi C. A.; Bazzanella N.; Patel N.; Miotello A. Rational Design Combining Morphology and Charge-Dynamic for Hematite/Nickel–Iron Oxide Thin-Layer Photoanodes: Insights into the Role of the Absorber/Catalyst Junction. ACS Appl. Mater. Interfaces 2019, 11 (51), 48002–48012. 10.1021/acsami.9b19790. 31797662

[ref49] Zandi O.; Klahr B. M.; Hamann T. W. Highly photoactive Ti-doped α-Fe_2_O_3_ thin film electrodes: resurrection of the dead layer. Energy Environ. Sci. 2013, 6 (2), 634–642. 10.1039/C2EE23620F.

[ref50] Upul Wijayantha K. G.; Saremi-Yarahmadi S.; Peter L. M. Kinetics of oxygen evolution at α-Fe_2_O_3_ photoanodes: a study by photoelectrochemical impedance spectroscopy. Phys. Chem. Chem. Phys. 2011, 13 (12), 5264–5270. 10.1039/c0cp02408b. 21229167

[ref51] Dias P.; Andrade L.; Mendes A. Hematite-based photoelectrode for solar water splitting with very high photovoltage. Nano Energy 2017, 38, 218–231. 10.1016/j.nanoen.2017.05.051.

