# Supplementary material for: Better Together: Ilmenite/Hematite Junctions for Photoelectrochemical Water Oxidation
Source: ACS Appl Mater Interfaces. 2020 Sep 28;12(42):47435–46. doi: 10.1021/acsami.0c12275 (PMC8014905; doi:10.1021/acsami.0c12275)
Supplement: Supplementary file 1 — am0c12275_si_001.pdf [file am0c12275_si_001.pdf]

Supporting information to:

Better Together: Ilmenite/Hematite Junctions for Photoelectrochemical Water Oxidation

Serena Berardi,[1] Jagadesh Kopula Kesavan,[2] Lucia Amidani,[3] Elia Marek Meloni,[1] Marcello Marelli,[4] Federico Boscherini,[2] Stefano Caramori,[1] Luca Pasquini[2,*]

1 - Department of Chemical and Pharmaceutical Sciences, University of Ferrara, via L. Borsari 46, 44121 Ferrara, Italy.
2 - Department of Physics and Astronomy, Alma Mater Studiorum – Università di Bologna, viale C. Berti Pichat 6/2, 40127 Bologna, Italy.
3 - Helmholtz-Zentrum Dresden-Rossendorf, c/o European Synchrotron Radiation Facility, 71 Avenue des Martyrs, 38000 Grenoble, France.
4 - CNR-SCITEC, Istituto di Scienze e Tecnologie Chimiche "Giulio Natta", Via G. Fantoli 16/15, 20138 Milano, Italy.

[*] corresponding author: luca.pasquini@unibo.it

CONTENT **page(s)**

Photoanodes preparation **S2**

Transient measurements **S3**

Electrochemical impedance spectroscopy **S3-S4**

Supplementary figures S1-S5 **S3-S8**

Supplementary tables S1-S3 **S9-S10**

Supplementary figures S6-S16 **S11-S18**

References **S19**

Photoanodes preparation.

Fluorine-doped Tin Oxide (FTO) slides (TEC 8 Ω/cm^2, Pilkington) were cleaned by 10 min sonication in an Alconox® aqueous solution, followed by 10 min sonication in isopropanol. The electrophoretic deposition of mesoporous hematite (MPH) films on FTO was adapted from previous reports.[1] Briefly, in a Teflon beaker $FeCl_3{\cdot}6H_2O$ (0.55 g, >99%, Sigma Aldrich) was dissolved in a mixed solution of ethanol (20 mL) and water (5 mL) containing sodium acetate (0.8 g, ≥98%, Sigma Aldrich), and kept in a steel autoclave at 180 °C for 12 h. The resulting red powder (iron oxide nanoparticles) was washed several times with water and acetone and then suspended in acetone (50 mL). An aliquot (5 mL) of this colloidal dispersion was then mixed with a solution containing iodine (20 mg, Sigma Aldrich, ≥99.8%) in acetone (45 mL), and sonicated for 10 minutes. Two FTO slides (2 x 3 cm) were mounted at a distance of 0.8 cm, immersed in the colloidal solution and polarized at 10 V for 35 s in two electrode configuration using a ECO Chemie Autolab PGSTAT 302/N potentiostat. A homogeneous coating of the iron oxide nanoparticles was obtained on the negative electrode, which was then washed with acetone and annealed at 550°C for 1 h in air, then ramped up to 800°C for 20 min, yielding the MPH photoanodes.

As regards the preparation of Ti-modified samples, the same procedure was used, but the proper amounts of a 10 mM titanium(IV) butoxide (Sigma Aldrich, 97%) solution in ethanol were added to the deposition dispersion just before the application of the 10 V potential. A nominal 5 and 10% concentration of Ti(IV) with respect to the molar concentration of iron (considering quantitative the conversion of $FeCl_3{\cdot}6H_2O$ in Fe_2O_3 nanoparticles during the hydrothermal growth) was introduced for the preparation of MPH_5Ti and MPH_10 Ti electrodes, respectively. Ethanol was also added to the deposition solution to keep the acetone/ethanol ratio at 49:1.

Some of the MPH_5Ti electrodes were further functionalized with an amorphous iron(III) oxyhydroxide (indicated as FeOEC) by means of 10 cycles of SILAR (Successive Ionic Layer Adsorption and Reaction) deposition. Each SILAR cycle consisted in the immersion of the electrode in a 50 mM $FeCl_3{\cdot}6H_2O$ aqueous solution for 10 seconds, followed by dipping (for further 10 seconds) in 0.1 M NaOH (Alfa Aesar, 98%) and rinsing with distilled water. After the 10 SILAR cycles, the electrode was annealed in air at 200°C for 20 min.

Transient measurements

Nanosecond Transient Absorption spectra (TAS) were obtained by irradiating the photoanodes with a *Continuum Surelite II* Nd:YAG laser (1064 nm, FWHM = 6-8 ns) at 355 nm (tripled frequency) as the pump pulse, while the probe source was provided by an *Applied Photophysics Xe lamp* (150 W), equipped with an *Action SpectraPro 2300i* monocromator (150 grating) and a *Hamamatsu R3896* photomultiplier tube detector. By acting on the Q switch, voltage supply and optically defocusing the laser beam, the excitation fluence was tuned to ca. 500 μJ/cm^2/pulse. The photoanodes, oriented at 45° with respect to both the pump and the probe sources, were placed in a quartz cell containing 0.1 M KOH (pH 13.3). Transient difference spectra were collected both under open circuit potential (V_{oc}) and under anodic bias in a two-electrode configuration using a Pt wire as counter electrode and a PGSTAT 101 potentiostat as the current/voltage source. The resulting transient traces were averaged over 100 laser pulses and corrected for the baseline (i.e. the average trace in the absence of the laser pulse). The ΔOD values at different time delays were plotted versus the wavelength to yield the TAS spectra. The measurements were collected using an oscilloscope impedance of 1 MΩ.

Transient Photocurrent experiments (TPC) were performed by irradiating the samples with the 355 nm harmonic of the same nanosecond Nd:YAG laser, attenuated by several neutral filters to yield an intensity of 500 μJ/cm^2/pulse. The photoanodes were placed in a three-compartment cell and immersed in 0.1 M KOH (pH 13.3). For the TPC measurements in the presence of a white light bias, the additional continuous irradiation (from the front side of the electrode) was provided by an ABET solar simulator, equipped with an AM 1.5 G filter and calibrated to 0.4 W/cm^2. Polarization of the photoanodes was achieved through chronoamperometric measurements sampled at 10^{-4} s intervals, using a PGSTAT101 potentiostat.

Electrochemical impedance spectroscopy

The photoelectrochemical water oxidation efficiency (η_{WO}) of the different photoanodes can be calculated using the resistance and capacitance values obtained from the fit of EIS data, according to literature reports.[2,3] Being related to the interfacial performances of the photoelectrodes under illumination, the η_{WO} depends on: (i) the kinetics

of water oxidation (i.e. the reaction of the surface trapped holes with the surface bound water molecules), to which a rate constant accounting for the charge transfer to the electrolyte (k_{CT}) is associated; (ii) the e^-/h^+ recombination rate (k_{REC}) at the surface of the hematite electrode, which depends on the applied bias, and (as far as the hole concentration is concerned) on the excitation intensity; (iii) the charge recombination in the bulk of the film (since this aspect does not significantly change upon surface modifications, it will be neglected in this method).

Both k_{CT} and k_{REC} can be extracted from the fit of EIS data. In particular, the former can be calculated as follows:

$$k_{CT} = (R_{CT,SS} \cdot C_{CT})^{-1}$$

where $R_{CT,SS}$ is the resistance of the charge transfer from the surface states to the electrolyte and C_{CT} is the space charge capacitance of hematite, obtained from the corresponding constant phase element (CPE_{CT}) admittance (extracted from the fit), according to the equation:

$$C = CPE \cdot (\omega)^{n-1}$$

being ω the angular frequency corresponding to the largest imaginary component of the charge transfer arc and n is the CPE exponent ($0.7 \leq n \leq 1$).

k_{REC} can instead be obtained by the following simplified equation, valid at high applied bias:[4]

$$k_{REC} \cong \frac{k_{CT} \cdot R_{CT,SS}}{R_{SC}} \quad ,$$

where R_{SC} is the charge transport resistance through the space charge.

Finally the photoelectrochemical water oxidation efficiency (η_{WO}) is calculated as:

$$\eta_{WO} = \frac{k_{CT}}{k_{CT} + k_{REC}} \ \% \quad .$$

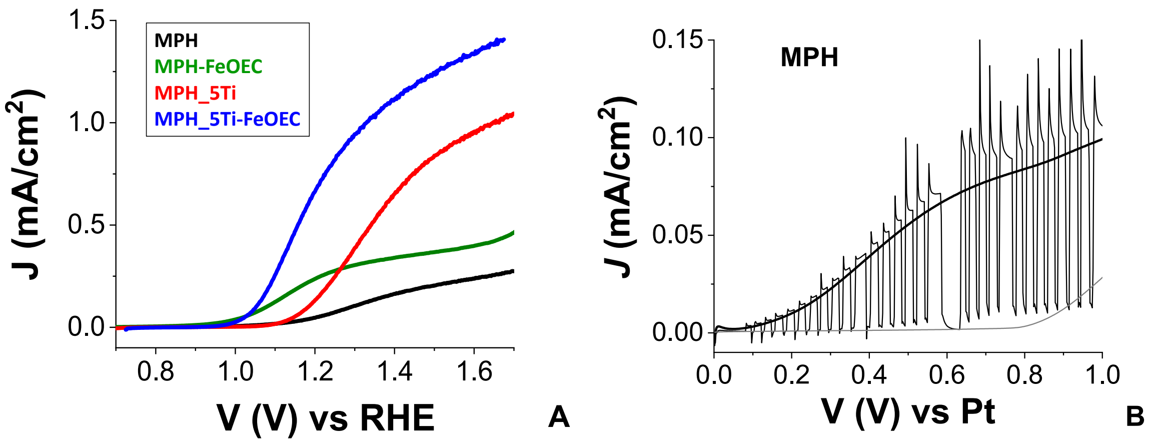

Figure S1. *(A) J-V curves of the MPH (black), MPH_5Ti (red) and MPH_5Ti-FeOEC (blue) photoanodes collected under 0.1 W/cm*^{2} *AM1.5G back illumination in 0.1 M KOH (pH 13.3). The J-V performances of a MPH-FeOEC photoanode is also reported for the sake of comparison (green curve). (B) J-V curves of the MPH photoanode collected under continuous or chopped illumination (0.1 W/cm*^{2} *AM1.5G) in 0.1 M KOH (pH 13.3) in 2-electrode configuration. The dark trace is also reported in gray.*

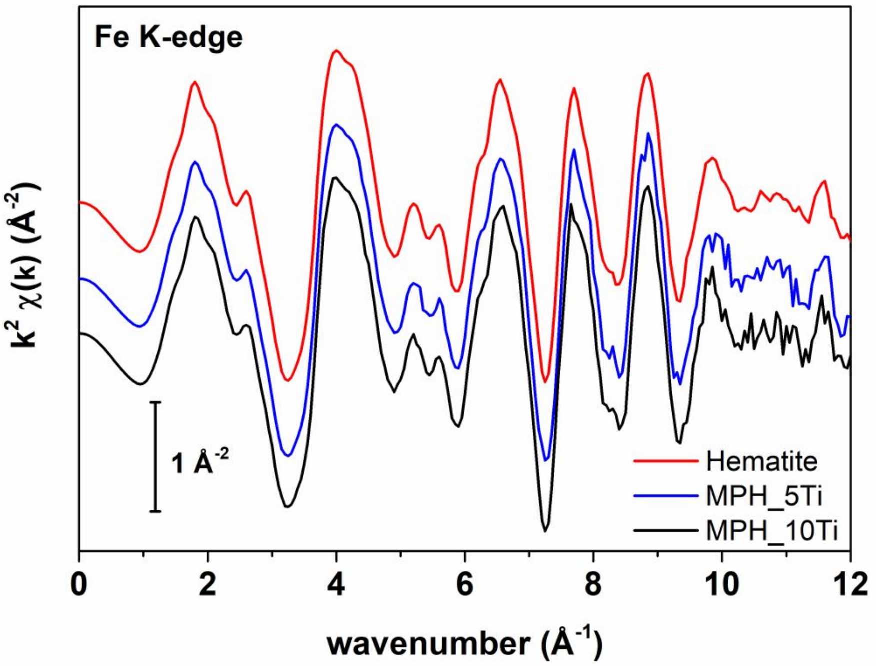

Figure S2. *Fe K edge EXAFS (k^2 weighted) spectra of Ti-modified MPH and reference hematite. Spectra have been vertically offset for clarity.*

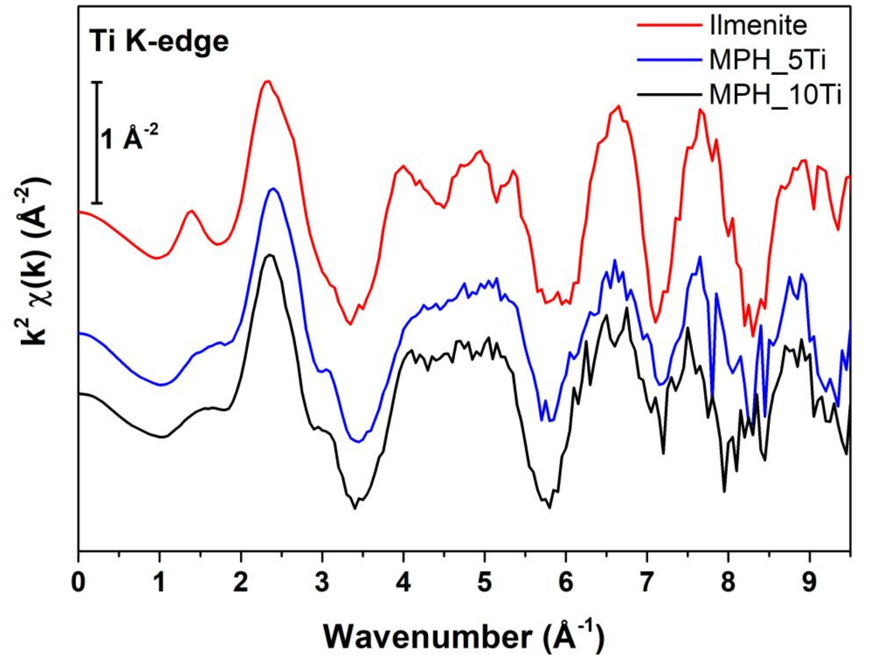

Figure S3. *Ti K edge (k^2 weighted) EXAFS spectra of Ti-modified MPH and reference hematite. Spectra have been vertically offset for clarity.*

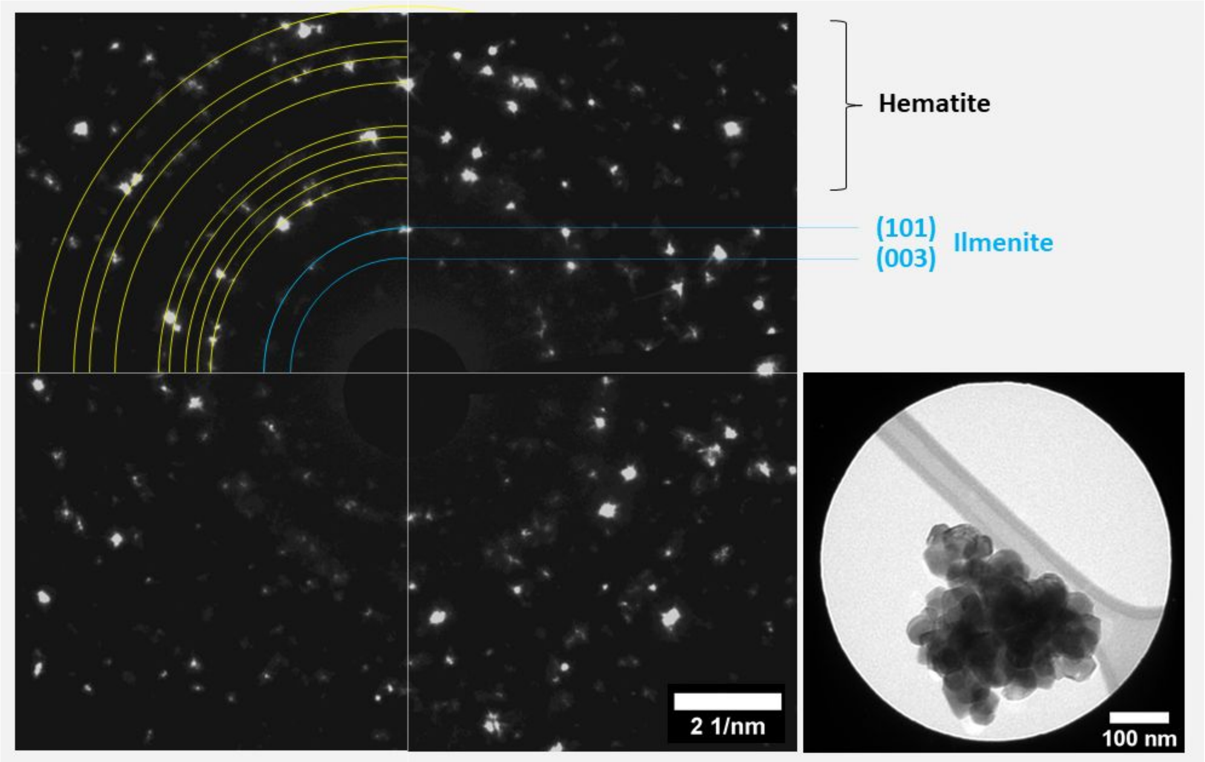

Figure S4. *SAED pattern of selected sample area (showed on the right side) and related indexing as hematite (yellow lines) and ilmenite (blue lines) crystal structures.*

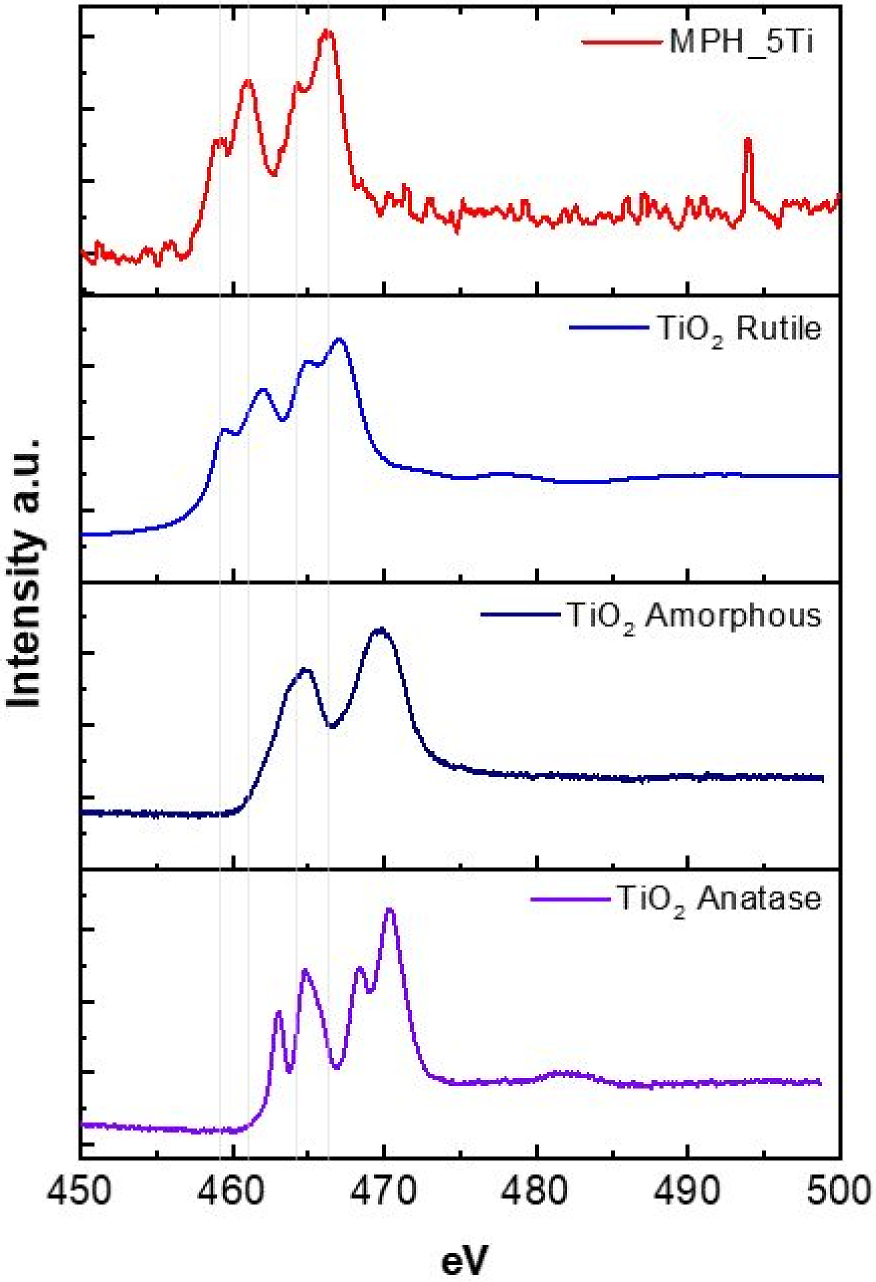

Figure S5. *Comparison of the EELS spectra for specimen MPH_5Ti (above) with those of TiO_2 reference samples (below) recoded under the same experimental conditions at the Ti $L_{2,3}$ edge. The peak positions and the overall profile shape do not match, suggesting that these TiO_2 phases are not present in the sample.*

Table S1. *Local structural parameters deduced from fitting of Fe K-edge EXAFS spectra. Uncertainties on the least significant figures are in parenthesis.*

**Sample**	**R-factor**	R_{Fe-O1}	σ^2_{Fe-O1}	R_{Fe-O2}	σ^2_{Fe-O2}	R_{Fe-Fe1}	σ^2_{Fe-Fe1}	R_{Fe-Fe2}	σ^2_{Fe-Fe2}	R_{Fe-Fe3}	σ^2_{Fe-Fe3}	R_{Fe-Fe4}	σ^2_{Fe-Fe4}
**MPH**	0.018	1.930 (15)	0.0049 (16)	2.088 (21)	0.0074 (29)	2.866 (35)	0.0028 (38)	2.975 (10)	0.0033 (10)	3.384 (15)	0.0045 (16)	3.687 (13)	0.009 (14)
**MPH_5Ti**	0.013	1.929 (11)	0.0058 (12)	2.080 (17)	0.0104 (25)	2.838 (22)	0.0053 (21)	2.959 (9)	0.0033 (8)	3.376 (8)	0.0050 (8)	3.677 (10)	0.0088 (9)
**MPH_10Ti**	0.019	1.935 (19)	0.0050 (21)	2.087 (27)	0.0082 (41)	2.860 (28)	0.0011 (27)	2.976 (14)	0.0023 (14)	3.381 (11)	0.0042 (11)	3.687 (14)	0.0084 (14)

Table S2*. Results of fitting analysis of Ti K pre-edge peaks. Uncertainties on the least significant figures are in parenthesis.*

	**Pre edge peak A1**			**Pre edge peak A2**			**Pre edge peak A3**			**Pre-edge peak B**			**A1 %**	**A2 %**	**A3 %**
**Sample**	**position**	**Height**	**HWHM**	**position**	**Height**	**HWHM**	**position**	**Height**	**HWHM**	**position**	**Height**	**HWHM**			
**Ilmenite**	4968.65 (33)	0.0589	0.73 (18)	4970.55 (10)	0.1560	0.70 (45)	4971.54 (40)	0.0830	1.10 (30)	4973.88 (90)	0.0383	1.00 (64)	17.60	44.70	37.70
**MPH_5Ti**	4968.85 (31)	0.0521	0.91 (16)	4970.47 (31)	0.0957	0.71 (52)	4971.27 (42)	0.1193	0.98 (19)	4973.79 (73)	0.0367	1.42 (61)	20.33	29.12	50.55
**MPH_10Ti**	4969.60 (62)	0.0598	1.49 (37)	4970.71 (25)	0.1018	0.66 (18)	4971.72 (77)	0.0863	0.87 (52)	4974.17 (37)	0.0498	1.59 (45)	38.50	29.00	32.50

***Table S3**. Local structural parameters deduced from fitting of Ti K-edge EXAFS spectra. Uncertainties on the least significant figures are in parentheses.*

**Sample**	**R-factor**	$R_{Ti\text{-}O1}$	σ^2_{Ti-O1}	$R_{Ti\text{-}O2}$	σ^2_{Ti-O2}	$R_{Ti\text{-}Ti1}$	σ^2_{Ti-Ti1}	$R_{Ti\text{-}Fe1}$	σ^2_{Ti-Fe1}	$R_{Ti\text{-}O3}$	σ^2_{Ti-O3}	$R_{Ti\text{-}Fe3}$	σ^2_{Ti-Fe3}
**Ilmenite**	0.0145	1.839 (30)	0.008 (4)	2.021 (43)	0.012 (7)	2.989 (16)	0.002 (1)	3.408 (40)	0.012 (6)	3.294 (27)	0.0202 (32)	3.760 (34)	0.012 (3)
**MPH_5Ti**	0.002	1.929 (67)	0.0154 (68)	2.038 (16)	0.0284 (24)	3.069 (31)	0.0080 (43)	3.465 (61)	0.0076 (67)	3.164 (87)	0.0305 (26)	3.710 (76)	0.0090 (56)
**MPH_10Ti**	0.015	1.938 (36)	0.0069 (32)	2.155 (15)	0.0346 (42)	3.077 (42)	0.0064 (45)	3.465 (33)	0.0208 (46)	3.264 (76)	0.0315 (45)	3.740 (26)	0.0172 (22)

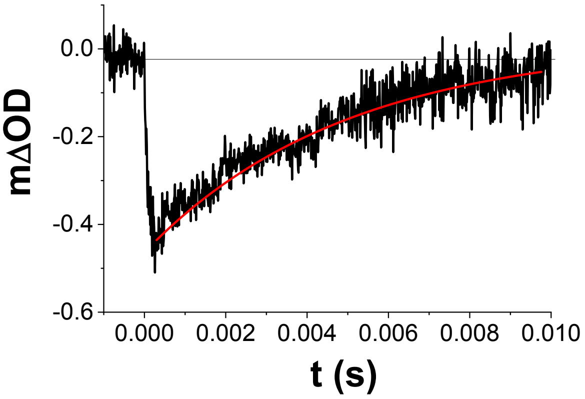

A

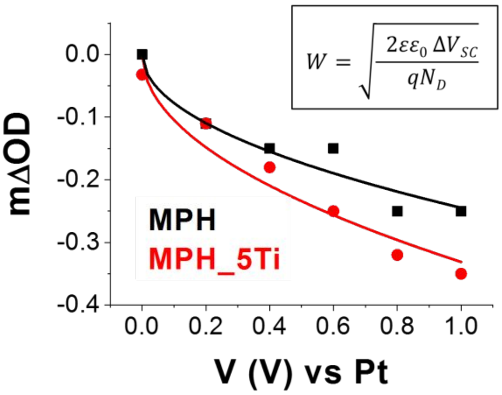

B

Figure S6. *(A) TAS trace of the 580 nm signal for MPH at 0.8 V vs Pt, evidencing the complete recovery of the bleach within 10 ms; (B) mΔOD values for the TAS traces recorded at 580 nm for both MPH and MPH_5Ti under different applied biases in 0.1 M KOH. 1 MΩ oscilloscope resistance. Inset: Gärtner's equation for the depletion layer width (W) as a function of the potential drop across the space charge (ΔV_{SC}). In the equation, ε represents the dielectric constant of the material, ε_0 the vacuum permittivity, q the electron charge and N_D the donor density.*

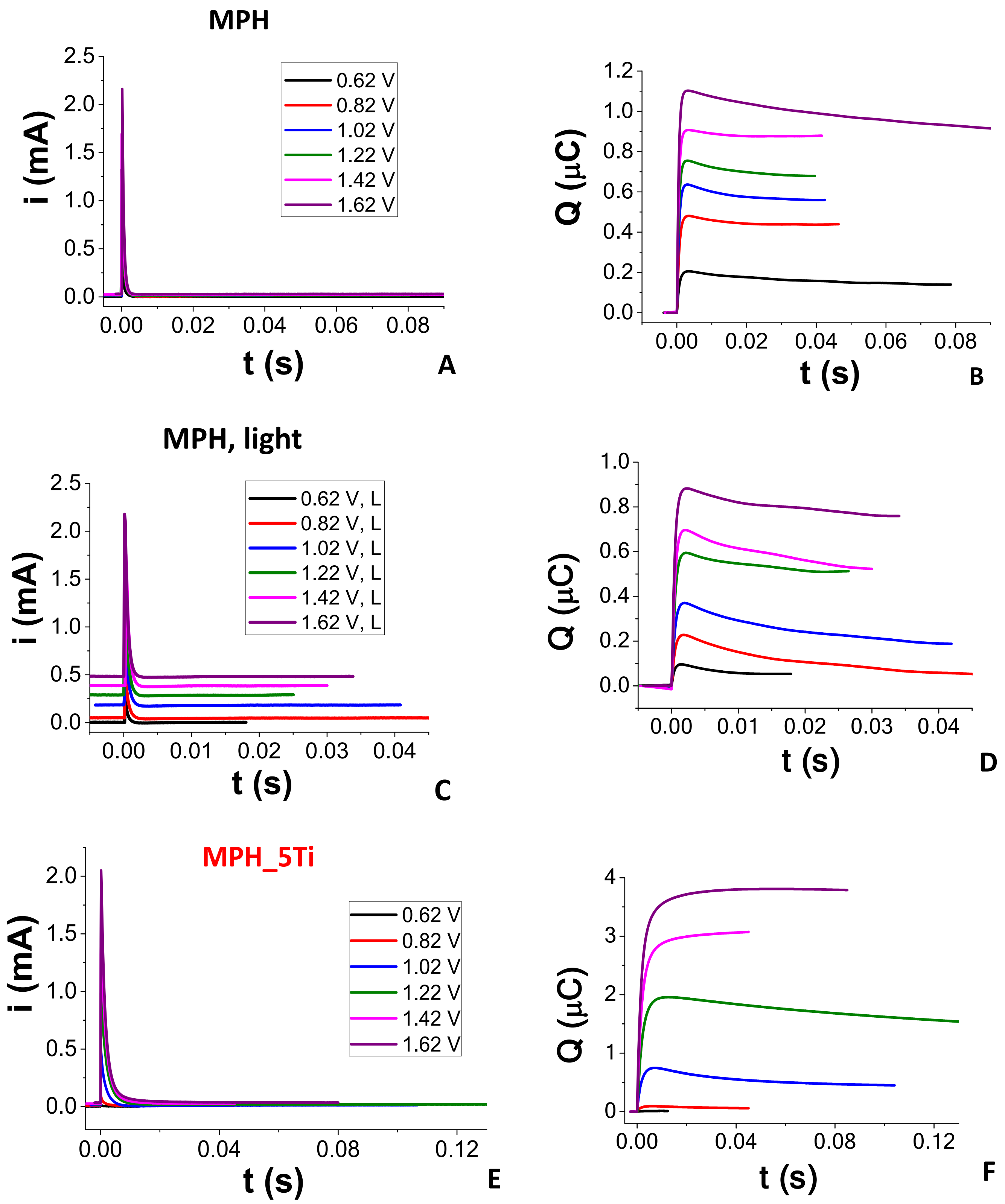
MPH
i (mA)
t (s)
0.62 V
0.82 V
1.02 V
1.22 V
1.42 V
1.62 V
A
Q (μC)
t (s)
B
MPH, light
i (mA)
t (s)
0.62 V, L
0.82 V, L
1.02 V, L
1.22 V, L
1.42 V, L
1.62 V, L
C
Q (μC)
t (s)
D
MPH_5Ti
i (mA)
t (s)
0.62 V
0.82 V
1.02 V
1.22 V
1.42 V
1.62 V
E
Q (μC)
t (s)
F

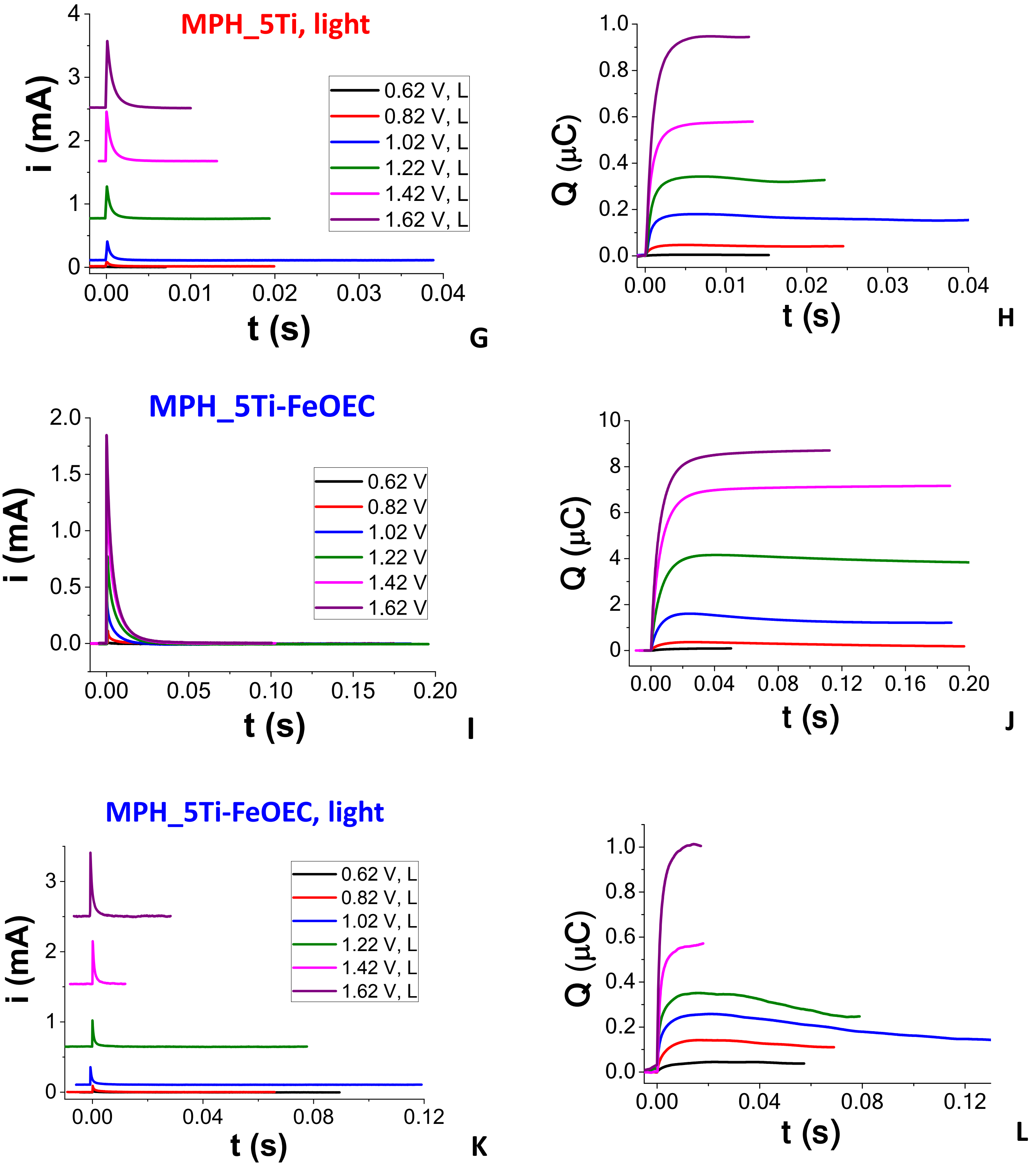

Figure S7. *Transient photocurrent decay traces and photogenerated charges obtained from their integration over time for MPH (A-D), MPH_5Ti (E-H) and MPH_5Ti-FeOEC (I-L) photoanodes. All the traces are recorded after the 355 nm ns-laser excitation, in the absence (A-B, E-F, I-J) or in the presence (C-D, G-H, K-L) of an additional white light source, under different applied biases.*

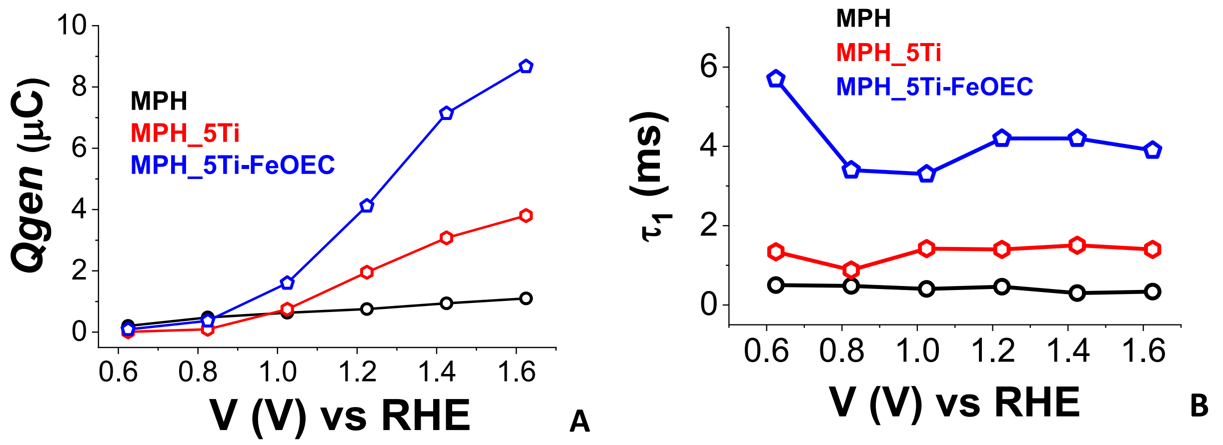

Figure S8*. Applied bias dependence of Qgen (A) and τ_1 (B) for MPH (black), MPH_5Ti (red) and MPH_5Ti-FeOEC (blue) photoanodes.*

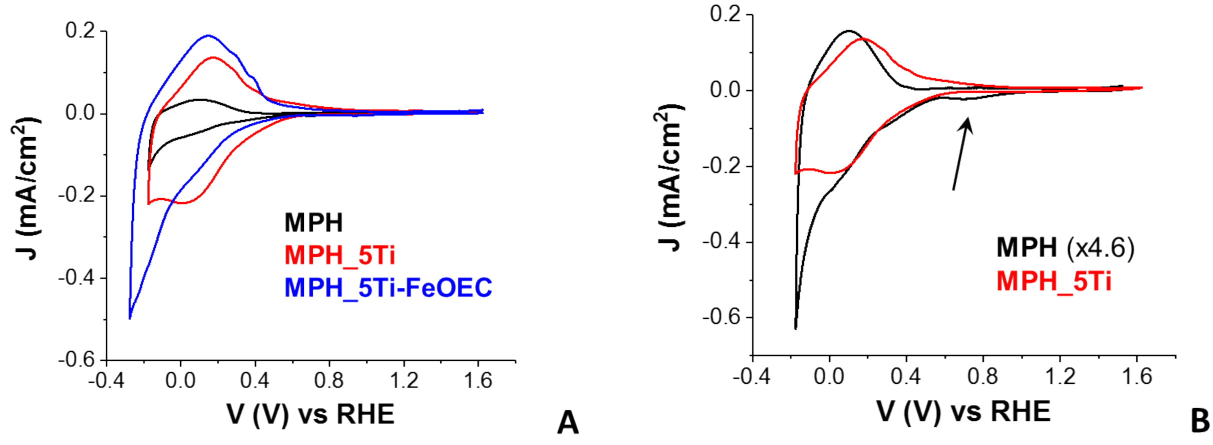

Figure S9*. (A) Cyclic voltammetry of MPH (black), MPH_5Ti (red) and MPH_5Ti-FeOEC (blue) recorded after a pre-conditioning step at 1.6 V for 120 s. The potential was then scanned from 1.6 V towards cathodic potentials (until reaching the threshold of the conduction band edge of the semiconductor), and then backwards to the start potential. The measurements were carried out in 0.1 M KOH under nitrogen stream, dark conditions, 20 mV/s scan rate. (B) Comparison of MPH (black) and MPH_5Ti (red) on the same current density scale, achieved by normalizing the J values of the two electrodes at 0.2 V (i.e. multiplying the CV curve of MPH by a factor 4.6). The arrow indicates the broad pre-wave at ca. 0.7 V in the unmodified MPH, which is suppressed in the MPH_5Ti electrode.*

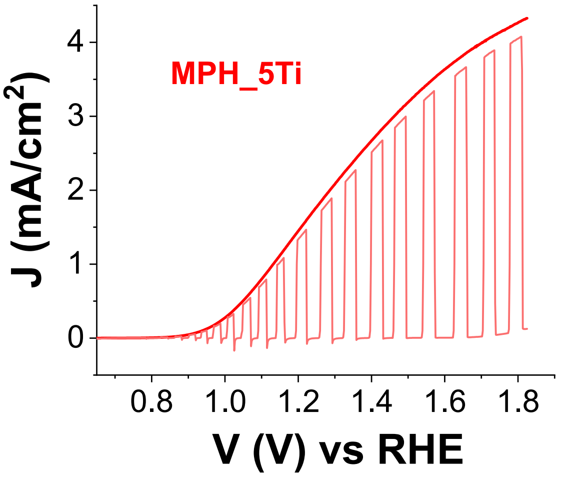

***Figure S10**. J-V curves of the MPH_5Ti photoanode collected under 0.4 W/cm^2 AM1.5G illumination.*

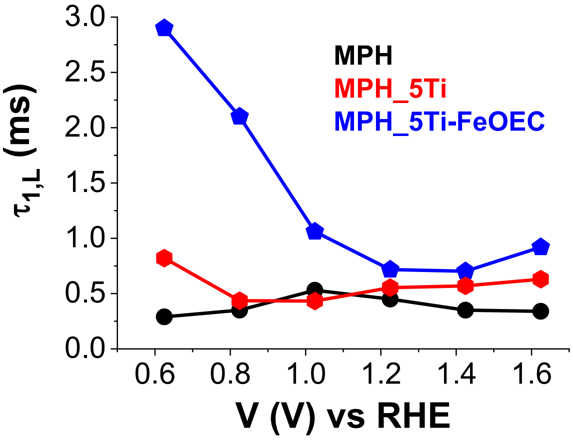

***Figure S11**. $\tau_{1,L}$ dependence on the applied bias.*

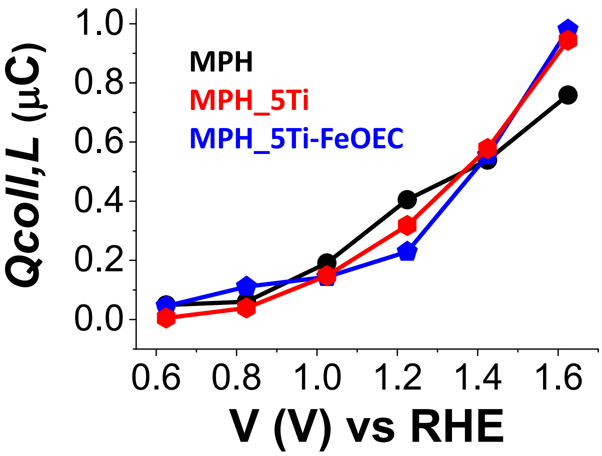

***Figure S12**. Applied bias dependence of Qcoll,L values in the presence of an additional white light bias provided by a solar simulator.*

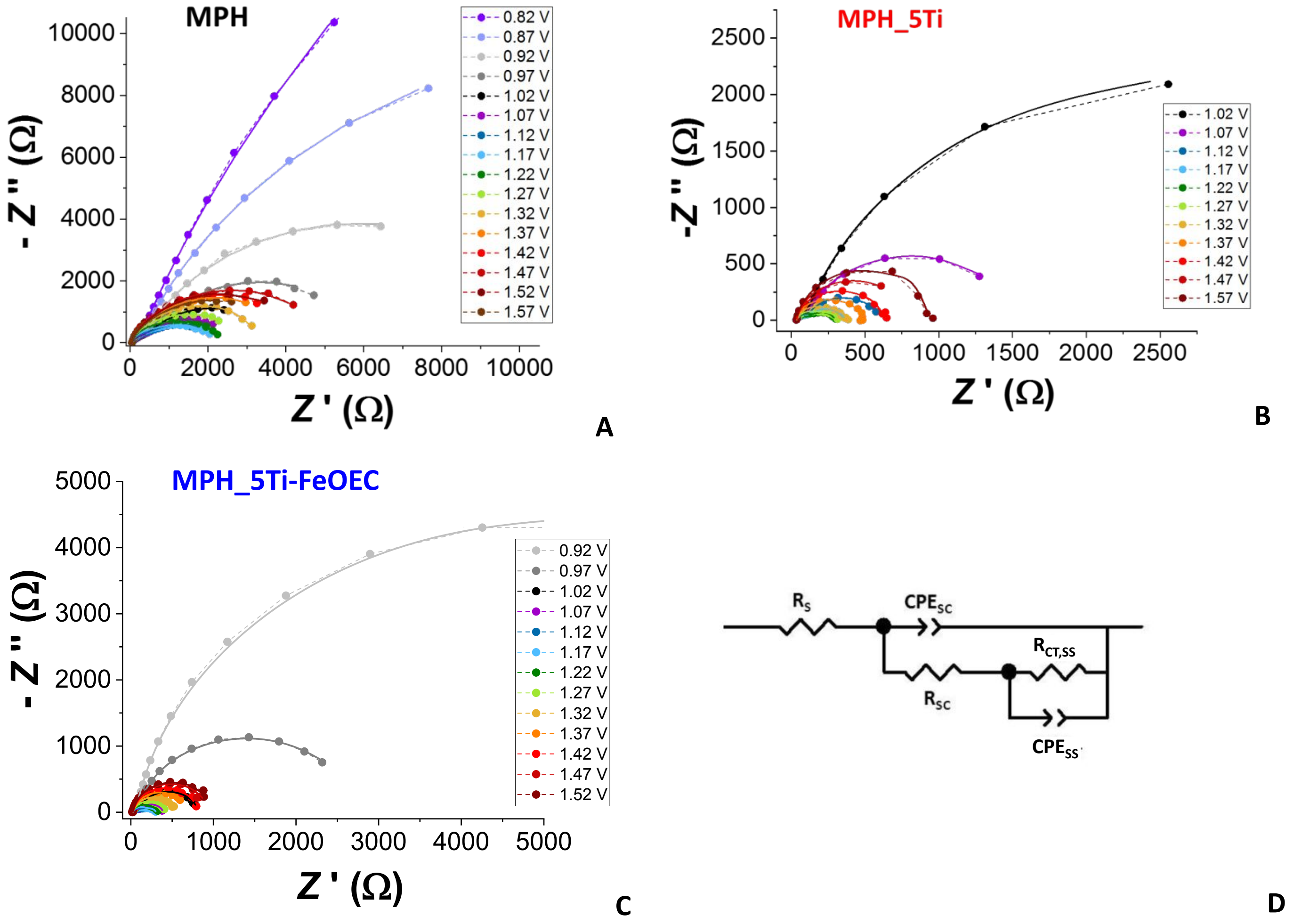

***Figure S13**. Complex plane Nyquist plots for MPH (A), MPH_5Ti (B) and MPH_5Ti-FeOEC (C) photoanodes, recorded in 0.1 M KOH (pH 13.3) under 1 sun (0.1 W/cm^2 AM1.5G) illumination and different applied biases (all values vs RHE). The corresponding fits are reported as solid lines. (D) Equivalent circuit used to fit the EIS data of the photoanodes. The different circuital elements included are described in the main paper. The constant phase elements (CPEs, i.e. non-ideal capacitances) can be converted in capacitance (C) values as indicated in page S4.*

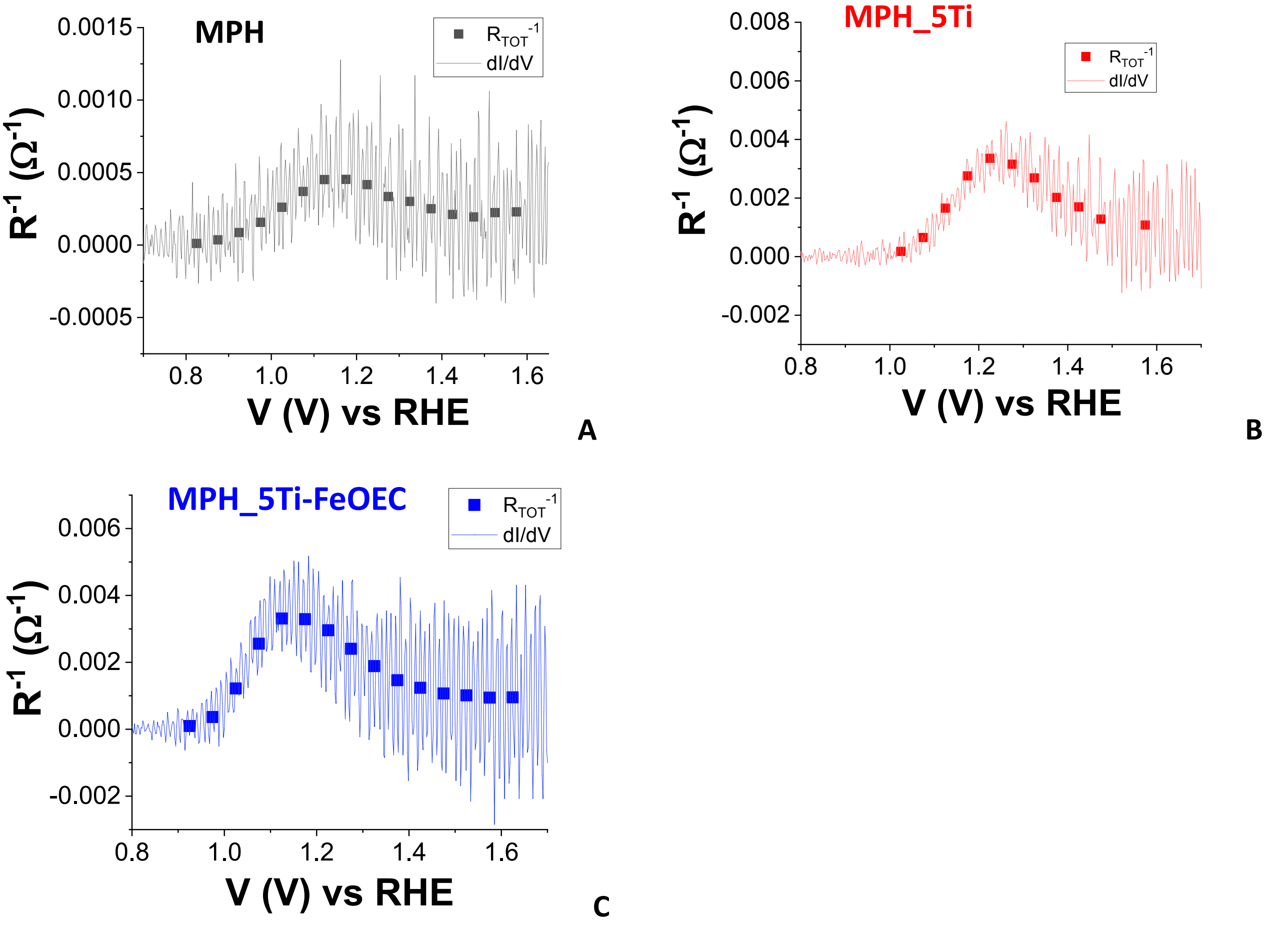

***Figure S14**. Applied bias dependence of R_{TOT}^{-1} (squares) for MPH (A), MPH_5Ti (B) and MPH_5Ti-FeOEC (C) photoanodes recorded in 0.1 M KOH (pH 13.3) under 1 sun (0.1 W/cm^2, AM1.5G) illumination. The corresponding derivatives of the I-V curves (dI/dV) are also reported as solid lines.*

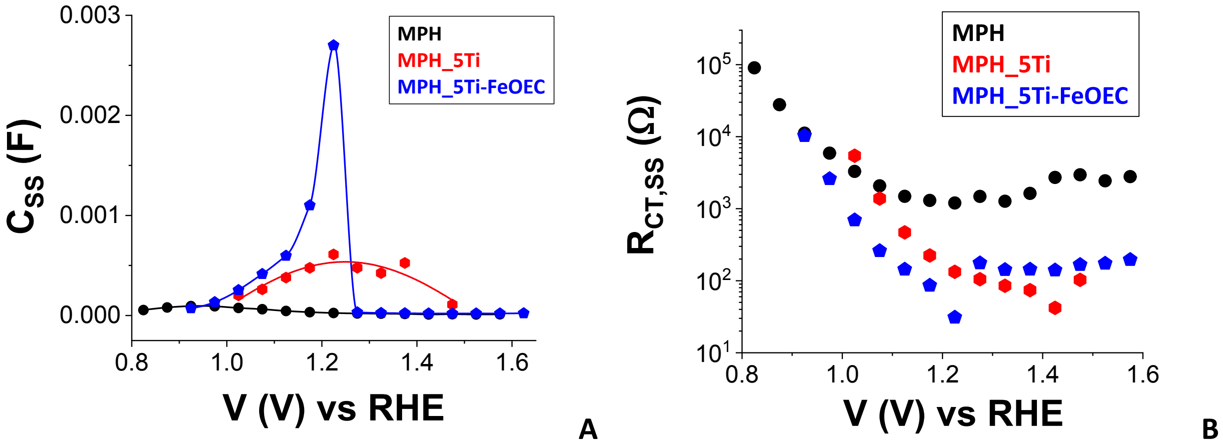

Figure S15. *Applied bias dependence of C_{SS} (A) and $R_{CT,SS}$ (B) for MPH (black), MPH_5Ti (red) and MPH_5Ti-FeOEC (blue) photoanodes recorded in 0.1 M KOH (pH 13.3) under 1 sun (0.1 W/cm^2, AM1.5G) illumination. The C_{SS} and $R_{CT,SS}$ values were extracted from the fit of EIS data.*

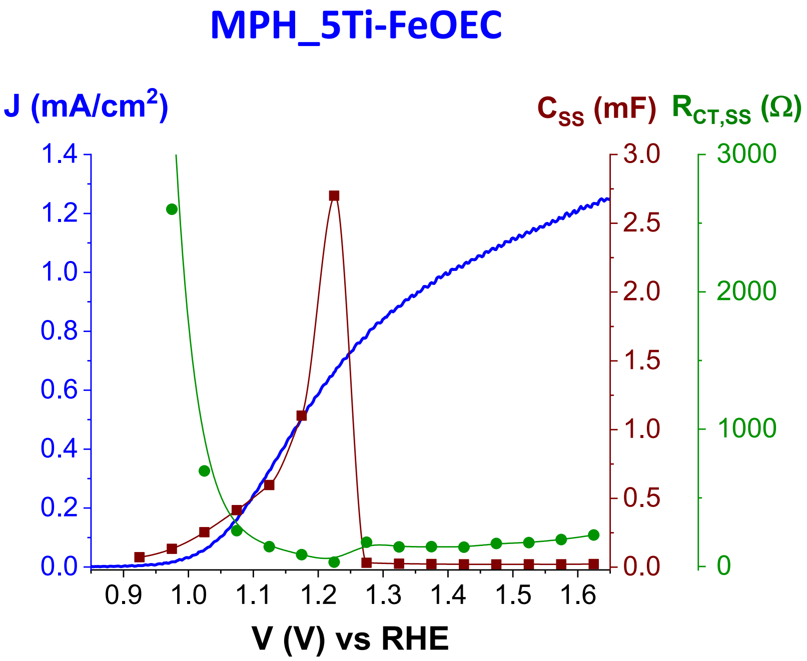

Figure S16. *Correlation between C_{SS} (wine) and $R_{CT,SS}$ (green) for a MPH_5Ti-FeOEC photoanode as a function of the applied bias. The J-V curve (blue trace) is also reported for the sake of comparison. All measurements were performed in 0.1 M KOH (pH 13.3) under 1 sun illumination (0.1 W/cm^2 AM1.5G).*

References

[1] Zong, X.; Thaweesak, S.; Xu, H.; Xing, Z.; Zou, J.; Lua, G. (M.); Wang, L. *Phys. Chem. Chem. Phys.* **2013**, *15*, 12314-12321.

[2] Upul Wijayantha, K. G.; Saremi-Yarahmadi, S.; Peter, L. M. *Phys. Chem. Chem. Phys.* **2011**, *13*, 5264−5270.

[3] Dias, P.; Andrade, L.; Mendes, A. *Nano Energy* **2017**, *38*, 218−231.

[4] Bertoluzzi, L.; Bisquert, J. *J. Phys. Chem. Lett.* **2012**, *3*, 2517−2522.
